# Prevalence of occult hepatitis B virus infection in adults: a systematic review and meta-analysis

**DOI:** 10.1016/S2468-1253(22)00201-1

**Published:** 2022-08-10

**Authors:** Yu Ri Im, Rukmini Jagdish, Damien Leith, Jin Un Kim, Kyoko Yoshida, Amir Majid, Yueqi Ge, Gibril Ndow, Yusuke Shimakawa, Maud Lemoine

**Affiliations:** a Section of Hepatology, Division of Digestive Diseases, Department of Metabolism, Digestion and Reproduction, Imperial College London, London, UK; b Department of Medicine, Chelsea and Westminster Hospital, London, UK; c Department of Gastroenterology, Chelsea and Westminster Hospital, London, UK; d Department of Gastroenterology, Glasgow Royal Infirmary, Glasgow, UK; e Royal Free Hospital, London, UK; f Faculty of Medicine, Tokyo Medical and Dental University, Tokyo, Japan; g Department of Anaesthetics, West Middlesex University Hospital, London, UK; h MRC Unit The Gambia at London School of Hygiene & Tropical Medicine, Banjul, The Gambia; i Unité d'Épidémiologie des Maladies Émergentes, Institut Pasteur, Paris, France

## Abstract

**Background:**

Despite growing concerns about transmissibility and clinical impact, occult hepatitis B virus (HBV) infection has received little attention in the hepatitis elimination agenda. We aimed to estimate the prevalence of occult HBV infection at a global and regional scale and in specific populations.

**Methods:**

For this systematic review and meta-analysis, we searched the MEDLINE, Embase, Global Health, and Web of Science databases for articles published in any language between Jan 1, 2010, and Aug 14, 2019. We included original articles and conference abstracts of any study design that reported the proportion of HBsAg-negative adults (aged ≥18 years) who are positive for HBV DNA (ie, people with occult HBV infection). The prevalence of occult HBV infection was pooled, using the DerSimonian-Laird random-effects model, in the general population and specific groups defined by the type of study participants (blood donors; other low-risk populations; high-risk populations; and people with advanced chronic liver disease), and stratified by HBV endemicity in each country. We also assessed the performance of anti-HBc as an alternative biomarker to detect occult HBV infection. The study was registered with PROSPERO, CRD42019115490.

**Findings:**

305 of 3962 articles were eligible, allowing a meta-analysis of 140 521 993 individuals tested for HBV DNA. Overall, only two studies evaluated occult HBV infection in the general population, precluding unbiased global and regional estimates of occult HBV infection prevalence. In blood donors, occult HBV infection prevalence mirrored HBV endemicity: 0·06% (95% CI 0·00–0·26) in low-endemicity countries, 0·12% (0·04–0·23) in intermediate-endemicity countries, and 0·98% (0·44–1·72), in high-endemicity countries (p=0·0012). In high-risk groups, occult HBV infection prevalence was substantial, irrespective of endemicity: 5·5% (95% CI 2·9–8·7) in low-endemicity countries, 5·2% (2·5–8·6) in intermediate-endemicity countries, and 12·0% (3·4–24·7) in high-endemicity countries. The pooled sensitivity of anti-HBc to identify occult HBV infection was 77% (95% CI 62–88) and its specificity was 76% (68–83).

**Interpretation:**

A substantial proportion of people carry occult HBV infection, especially among high-risk groups across the globe and people living in highly endemic countries. Occult HBV infection should be part of the global viral hepatitis elimination strategy.

**Funding:**

None.

## Introduction

Hepatitis B virus (HBV) infection is a major global health burden. In 2019, an estimated 296 million people were chronically infected with HBV and more than 820 000 HBV-related deaths occurred worldwide.^1^ Only 10·5% of chronically infected people were aware of their infection in 2019,^2^ suggesting a pressing need for more effective strategies to identify and treat individuals who are infected. The WHO clinical guidelines advocate initial testing with HBsAg using laboratory-based immunoassays or rapid diagnostic tests.^3^ This approach also applies to high-risk populations, such as people living with HIV, people with hepatitis C virus infection, and people on haemodialysis, and those with advanced chronic liver disease of unknown aetiology.^3^

However, these testing strategies are at risk of missing occult HBV infection, defined as the presence of replication-competent HBV DNA in the liver tissue or blood of individuals who have tested negative for HBsAg using chemiluminescent immunoassay or ELISA.^4^ HBV DNA should be detected by nucleic acid tests (NAT), including PCR, in blood samples or liver tissue, and the gold standard remains testing for episomal covalently closed circular DNA in the liver.^4^

The pathophysiology of occult HBV infection is not well characterised. HBsAg might become negative following the resolution of acute or chronic HBV infection, while HBV DNA is still detectable (post-window period). In such cases, the viral load tends to be low, usually less than 200 international units [IU]/mL.^5^ Other mechanisms include variants in HBsAg (S-variants or so-called S-escape mutations), which result in the HBsAg not being recognised by widely available HBsAg assays,^6^ or pre-S1 or pre-S2 variants that affect the expression of HBsAg.^7^, ^8^ It is postulated that these patients have circulating HBV DNA concentrations similar to those with chronic HBV infection who carry HBsAg detectable by the same HBsAg assays.^4^

**Research in context**

**Evidence before this study**

Occult hepatitis B virus (HBV) infection is defined as the presence of replication-competent HBV DNA in the blood or liver of individuals who test negative for HBsAg. Emerging evidence suggests that HBV might be transmitted from individuals carrying occult HBV infection through blood transfusion, and people with occult HBV infection might be at increased risk of cirrhosis and hepatocellular carcinoma. However, its global burden has not been estimated. We searched PubMed for articles published from inception to Jan 14, 2022, in any language, using the terms “systematic review” AND “occult hepatitis B” AND “prevalence”. Our search identified two articles. One article is a descriptive systematic review by Huang and Hollinger, published in 2014, on the risk of hepatocellular carcinoma in people with occult HBV infection, which did not estimate the prevalence of occult HBV infection in any population as an outcome. The second article is a systematic review and meta-analysis of occult HBV infection prevalence in Sudan, which estimated the prevalence in this country as 15·5%, but did not consider occult HBV infection prevalence elsewhere or at a global scale.

**Added value of this study**

The comprehensive search in our study identified 305 eligible articles, allowing a meta-analysis of 140 521 993 people. However, there were only two population-based surveys targeting the general population, precluding global and regional estimates of occult HBV infection prevalence in the general population. In subgroup analyses, occult HBV infection was frequent in specific groups, especially in people living in countries with a high prevalence of HBsAg and in high-risk populations irrespective of HBsAg prevalence in each country. We noted a large degree of heterogeneity even after stratifying the analysis by the type of study participants and HBV endemicity in the country of study (range of *I*^2^=55–98%). Access to HBV DNA measurement remains a major obstacle to diagnosing occult HBV infection in resource-limited countries, which are the most affected by the HBV epidemic worldwide. Therefore, we assessed the performance of anti-HBc to identify occult HBV infection. The pooled sensitivity was 77% (95% CI 62–88) and the pooled specificity was 76% (68–83).

**Implications of all the available evidence**

The WHO hepatitis elimination strategy does not currently consider occult HBV infection as a target for diagnosis and elimination. Yet the high prevalence of occult HBV infection in some populations observed in our study and the risk of potential transmission via blood donations suggest that occult HBV infection should no longer be neglected. Detection of occult HBV infection will require access to appropriate testing facilities, including in resource-limited settings. Given the suboptimal sensitivity and specificity of anti-HBc to identify occult HBV infection, this serological marker is not a reliable alternative to nucleic acid testing. Taken together, our findings imply that HBV elimination plans should consider occult HBV infection as a global health issue and improve access to nucleic acid testing at a low cost or promote the development and use of reliable, straightforward, and inexpensive markers of HBV DNA.

Despite an increasing number of occult HBV infection studies, the worldwide prevalence patterns of occult HBV infection remain unknown and its clinical relevance has been controversial.^9^ However, emerging evidence is shifting scientific opinion.^5^ There is increasing evidence that occult HBV infection is associated with advanced chronic liver disease, especially hepatocellular carcinoma,^10^, ^11^ and that people with occult HBV infection can transmit HBV infection. In addition, an estimated 8–29% of recipients from blood donors with occult HBV infection are infected with HBV.^12^, ^13^ These findings suggest that the role of occult HBV infection on global elimination targets might be highly underestimated, especially in low-income and middle-income countries (LMICs), where HBV DNA measurement remains largely inaccessible, leading to undiagnosed occult HBV infection cases.

By contrast, antibody to HBV core antigen (anti-HBc), a marker of current or past HBV infection, is easier to detect using immunoassays and more accessible at a low cost in LMICs. Anti-HBc is often used as a surrogate marker to detect seropositive occult HBV infection (ie, occult HBV infection cases positive for anti-HBc), especially in blood banks in LMICs.^4^ However, the overall performance of anti-HBc to identify occult HBV infection cases has not been well investigated, and it is uncertain whether anti-HBc is a reliable alternative to HBV NAT to identify occult HBV infection.

The aims of this systematic review and meta-analysis were to evaluate: (1) the global and regional prevalence of occult HBV infection on the basis of studies of the general population; (2) the occult HBV infection prevalence in subgroups defined by the type of study participants and by the HBV endemicity in each country; (3) whether the estimates vary with methodological differences, including the analytical sensitivity of HBsAg or HBV DNA assays used; and (4) the sensitivity and specificity of anti-HBc to detect occult HBV infection.

## Methods

### Search strategy and selection criteria

For this systematic review and meta-analysis, we searched the MEDLINE, Embase, Global Health, and Web of Science databases for articles evaluating occult HBV infection prevalence published between Jan 1, 2010, and Aug 14, 2019, using the following search terms: “hepatitis B” AND “occult” AND “prevalence” (see the appendix [pp 2–3] for the full search strategy). We chose this search period due to the introduction of more sensitive assays for HBsAg and HBV DNA.^14^ Following duplicate removal, DL and KY screened each entry to identify articles meeting inclusion criteria.

We included original articles and conference abstracts of any study design from which we could calculate the occult HBV infection prevalence, without any language restriction. We defined occult HBV infection prevalence as the proportion of HBsAg-negative adults (age ≥18 years) who had a positive result at any level for HBV DNA by NAT on blood samples or liver tissue, regardless of anti-HBc status. A viral load threshold of less than 200 IU/mL^5^ was not considered to define occult HBV infection, as most studies did not apply this cutoff. When acute window period infections (defined by detectable HBV DNA but undetectable HBsAg, which becomes detectable in later samples of the individual) were confirmed with subsequent samples from the same individual showing positivity for HBsAg, we excluded the individual or cohort. We included studies that systematically tested HBV DNA in people identified to be negative for HBsAg, irrespective of whether they had anti-HBc or antibodies against the HBV surface antigen (anti-HBs), and studies that tested HBV DNA in HBsAg-negative participants who were selected on the basis of anti-HBc or anti-HBs serostatus, or both. We excluded studies that only tested HBV DNA in less than 80% of HBsAg-negative individuals eligible for the occult HBV infection assessment without a clear explanation. We also excluded studies that included children or adolescents (aged <18 years) or individuals receiving nucleoside or nucleotide analogues or interferon therapy. For any such studies, we included them only if the authors provided individual data. If occult HBV infection prevalence was reported in more than one cohort, either due to different assays used on the same study populations or when the study included distinct population types (eg, HIV-positive and HIV-negative people), separate data were extracted for distinct cohorts (referred to as separate studies hereafter). By applying these eligibility criteria, we included different types of studies that answer different questions: (1) studies evaluating HBV DNA in HBsAg-negative people to estimate the occult HBV infection prevalence, and (2) studies evaluating both HBV DNA and anti-HBc in HBsAg-negative people to compute the diagnostic sensitivity and specificity of anti-HBc to indicate occult HBV infection.

### Data analysis

At least two of seven independent reviewers (YRI, RJ, DL, JUK, KY, AM, and YG) extracted the following data for each article: study settings, characteristics of participants, laboratory assays, and prevalence of occult HBV infection and anti-HBc. Discrepancies were settled by discussion with ML or YS.

First, we estimated occult HBV infection prevalence according to the sample type used to detect HBV DNA (serum or plasma, liver, or peripheral blood mononuclear cells [PBMCs]) and the serological criteria used to assess occult HBV infection (irrespective of anti-HBc or anti-HBs, or only those positive or negative for anti-HBc or anti-HBs). We assessed risk of bias using an adapted version of the tool developed by Hoy and colleagues.^15^ Second, by limiting studies to those that tested HBV DNA in serum or plasma irrespective of anti-HBc or anti-HBs, we estimated the global and regional prevalence in the general population as well as in specific population groups. We categorised the specific population groups as follows: blood donors; other low-risk populations (ie, general population, health-care workers, and pregnant women); high-risk populations (ie, people living with HIV, people with hepatitis C virus [HCV] infection, and people on haemodialysis); and people with advanced chronic liver disease (ie, cirrhosis or hepatocellular carcinoma).

We stratified population-specific estimates by country-level HBV endemicity (low: HBsAg prevalence <2·0%; intermediate: 2·0–4·9%; and high: ≥5·0%)^16^, ^17^ or by WHO regions (appendix pp 4–8). We also performed subgroup analyses by the serological criteria to test for occult HBV infection (eg, studies restricted to anti-HBc-positive individuals only, or anti-HBc-negative individuals only) and type of assays used to detect HBsAg or HBV DNA. The prevalence was pooled using the DerSimonian-Laird random-effects model after the variance of the proportions was stabilised by the Freeman-Tukey double arcsine transformation.^18^ We assessed heterogeneity using *I*^2^ statistics. To pool sensitivity and specificity of anti-HBc to diagnose occult HBV infection, we used the bivariate random-effects model,^19^ which considers the negative correlation between sensitivity and specificity to allow for the expected trade-off between these.^20^ Bivariate normality assumption was confirmed using χ^2^ probability plot of squared Mahalanobis distances (appendix p 16).^19^ Based on parameters derived by the bivariate random-effects model, we constructed a hierarchical summary receiver operating characteristic (HSROC) curve.^19^ Publication bias was assessed by plotting study size against the logarithm of the odds of prevalence.^21^ We used Stata/IC, version 16.1, for all analyses. We adhered to PRISMA guidelines and registered the study protocol in PROSPERO, CRD42019115490.

### Role of the funding source

There was no funding for this study.

## Results

**Figure 1:**
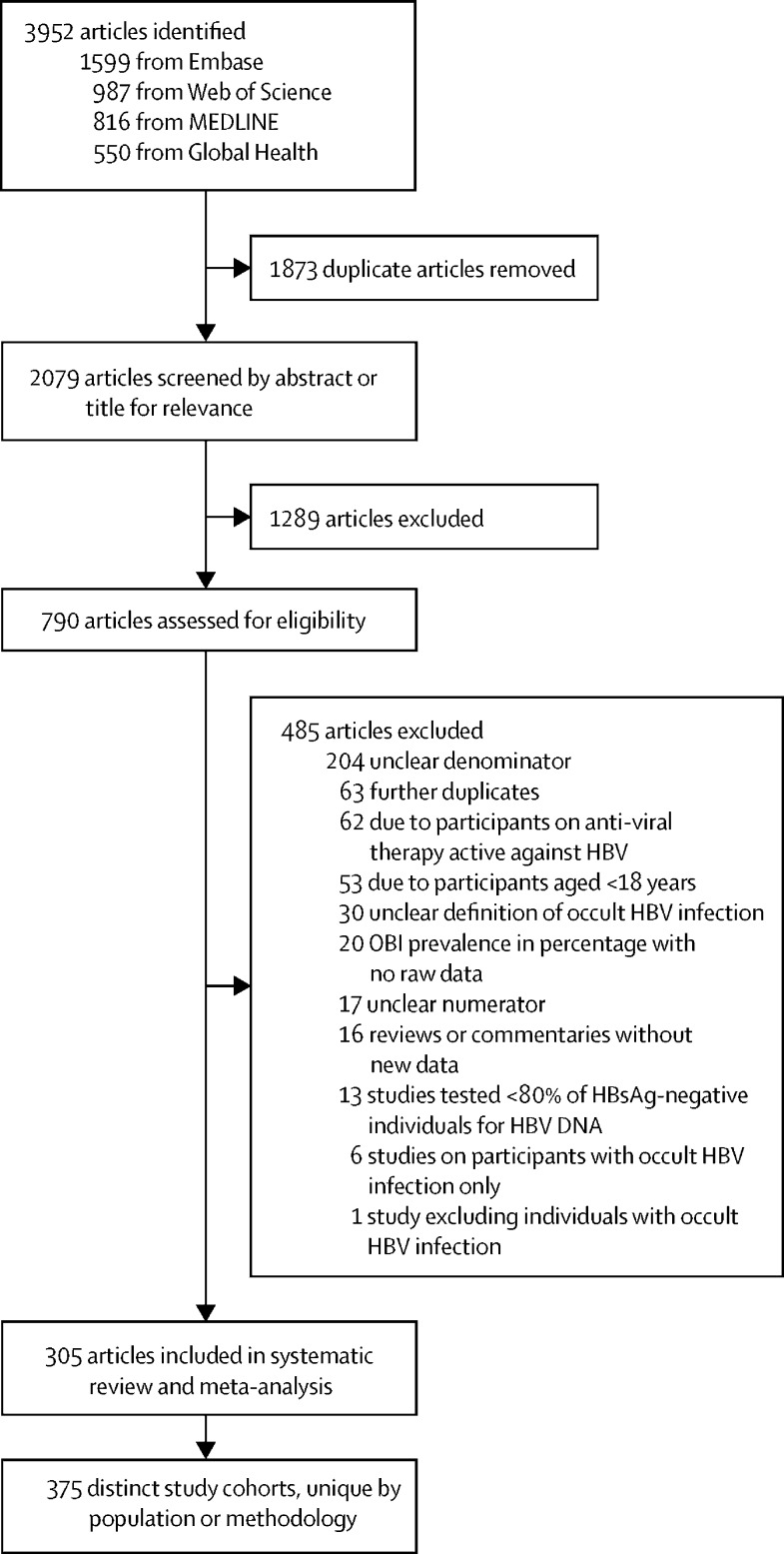
Study selection HBV=hepatitis B virus. OBI=occult HBV infection.

Of 3952 articles identified, 2079 were screened after duplicate removal and 305 met eligibility criteria, allowing a meta-analysis of 375 unique studies (figure 1; appendix pp 29–55). 140 521 993 HBsAg-negative individuals were tested for HBV DNA and served as a denominator for the prevalence of occult HBV infection (table). In terms of WHO regions, 121 (32%) of 375 studies were conducted in the Western Pacific Region, followed by 80 (21%) in the Eastern Mediterranean Region, 70 (19%) in the European Region, 45 (12%) in the Region of the Americas, 34 (9%) in the South-East Asian Region, and 24 (6%) in the African Region (table). The median of the mean age of the participants in each study was 49 years (IQR 37–55). The proportion of male participants in each study varied between 0% and 100%, with a median of 62% (IQR 51–76). In people with occult HBV infection, the median age was 51 years (40–63) and the proportion of male participants was 67% (50–89). The mean viral load in people with occult HBV infection was less than 200 IU/mL in 11 (55%) of 20 studies reporting the concentrations of HBV DNA. Among 78 studies reporting HBV genotypes in people with occult HBV infection, the distribution of these genotypes across the WHO regions mirrored the known distribution of HBV genotypes in HBsAg-positive people (appendix p 24). 336 (90%) of 375 studies used serum or plasma, 36 (10%) used liver tissue, and three (<1%) used PBMCs.

**Table:** Characteristics of the study population

	**Total number of studies**	Number of participants evaluated for occult HBV infection*	**Median of mean ages in individual studies**†	**Median of proportions of males in individual studies**‡
**Overall**				
All studies	375	140 521 993	49 (37–55)	62% (51–76)
**Publication year**				
2010–14	198 (53%)	69 452 684 (49%)	48 (37–56)	60% (50–75)
2015–19	177 (47%)	71 069 309 (51%)	49 (38–58)	62% (52–77)
**Population groups**				
Blood donors	133 (35%)	140 477 551 (>99%)	31 (30–35)	88% (77–95)
Other low-risk groups (ie, general population, healthy health-care workers, pregnant women)	19 (5%)	3840 (<1%)	38 (37–40)	34% (4–40)
High-risk populations due to risk of exposure (HIV, HCV, haemodialysis)	76 (20%)	11284 (<1%)	50 (45–55)	59% (49–64)
People with advanced chronic liver disease (ie, hepatocellular carcinoma or cirrhosis)	33 (9%)	1892 (<1%)	59 (54–65)	74% (68–79)
All other populations that did not fit into any of the above categories	114 (30%)	27 426 (<1%)	44 (36–52)	56% (50–73)
**WHO region**				
African Region	24 (6%)	453 673 (<1%)	37 (35–39)	45% (27–68)
Region of the Americas	45 (12%)	22 476 271 (16%)	49 (36–53)	58% (51–76)
Eastern Mediterranean Region	80 (21%)	178 909 (<1%)	46 (36–52)	65% (56–78)
European Region	70 (19%)	26 890 013 (19%)	49 (43–61)	59% (48–67)
South-East Asian Region	34 (9%)	1 147 862 (1%)	40 (32–45)	75% (56–89)
Western Pacific Region	121 (32%)	78 402 117 (56%)	57 (51–64)	60% (48–70)
Mixed	1 (<1%)	10 973 148 (8%)	NA	NA
**Endemicity**				
Low (HBsAg <2·00%)	186 (50%)	102 768 261 (73%)	49 (37–55)	63% (54–76)
Intermediate (HBsAg 2·00–4·99%)	78 (21%)	1 381 653 (1%)	45 (38–58)	63% (48–77)
High (HBsAg ≥5·00%)	106 (28%)	24 341 416 (17%)	50 (39–55)	54% (37–66)
Mixed countries	5 (1%)	12 030 663 (9%)	NA	NA

Data are n (%) or median (IQR). HBV=hepatitis B virus. HCV=hepatitis C virus. NA=not available.

* The number of participants evaluated for occult HBV infection using nucleic acid testing is a subset of the number of participants recruited to the study.

† Data on age were pooled from 92 studies that reported mean age for all recruited participants irrespective of HBsAg status, and 44 studies that only recruited known HBsAg-negative individuals and reported the mean age for these participants; data on age for the subset of participants evaluated for occult HBV infection using nucleic acid testing were very scarce and are therefore not shown here.

‡ Data on sex were pooled from 119 studies that reported sex for all recruited participants irrespective of HBsAg status, and 60 studies that only recruited known HBsAg-negative individuals and reported sex for these participants; data on age for the subset of participants evaluated for occult HBV infection using nucleic acid testing were very scarce and are therefore not shown here.

The pooled prevalence of occult HBV infection was 0·09% (95% CI 0·07–0·11; *I*^2^=99%) in 140 518 289 serum or plasma samples, 2·1% (0·0–10·1; *I*^2^=96%) in 1106 PBMC samples, and 34·8% (27·0–43·0; *I*^2^=94%) in 2598 liver samples. There was a wide variation in serological criteria used to test HBV DNA across the 375 studies, and in pooled prevalence of occult HBV infection according to these serological criteria: HBsAg-negative only (n=250 studies; <0·1%), HBsAg-negative and anti-HBc-positive (n=84; 9·9%), HBsAg-negative and anti-HBc-positive and anti-HBs-negative (n=26; 11·9%), HBsAg-negative and anti-HBc-negative and anti-HBs-negative (n=5; 7·4%), or HBsAg-negative and any other criteria (n=10; 9·8%; appendix pp 9–10). For the subsequent analyses, we only focused on studies that systematically tested HBV DNA using serum or plasma in HBsAg-negative people, irrespective of anti-HBc or anti-HBs serostatus, and excluded repeat donors, who might have a lower prevalence of occult HBV infection than first-time donors (appendix p 11).

Most studies estimated occult HBV infection prevalence in the specific populations: blood donors (133 [35%] of 375), pregnant women (three [1%]), health-care workers (seven [2%]), high-risk groups (76 [20%]), and people with advanced chronic liver disease (33 [9%]; table), with the remaining 123 studies (33%) assessing prevalence in other populations. There were only two studies (1%) estimating occult HBV infection prevalence in the general population regardless of anti-HBc serostatus.^22^, ^23^ One was a population-based serosurvey of 1007 individuals living in Canada, with the prevalence of occult HBV infection being 5·5%.^23^ Another was an epidemiological survey of 121 individuals in China, of whom four (3·3%) had occult HBV infection.^22^ Given the sparse number of studies targeting the general population, we could not estimate the global or regional prevalence of occult HBV infection.

**Figure 2:**
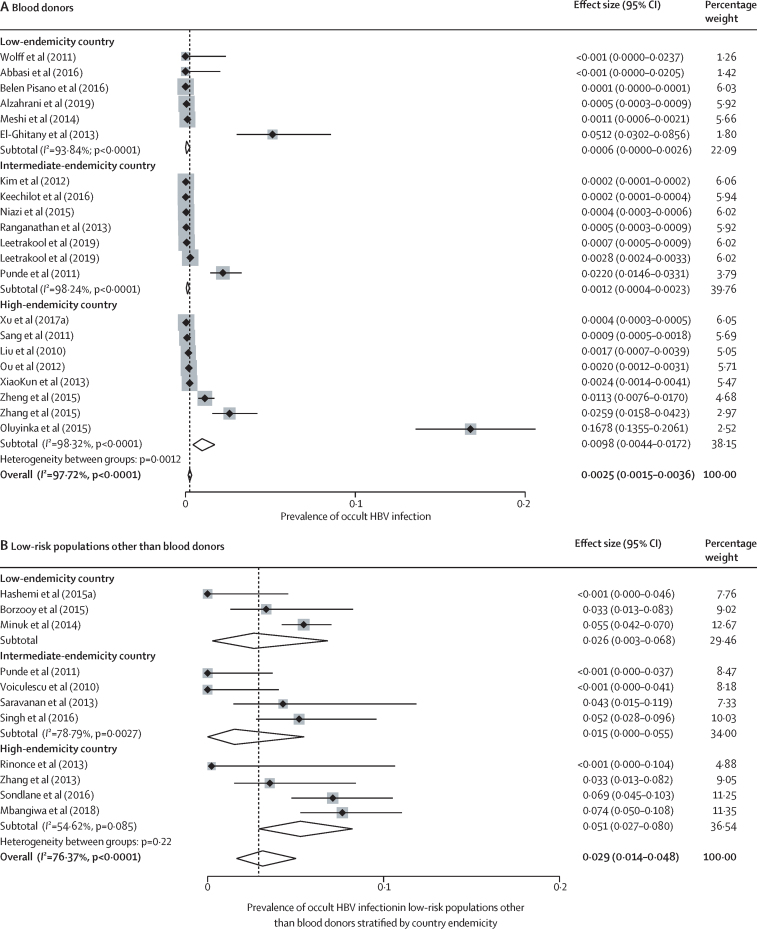

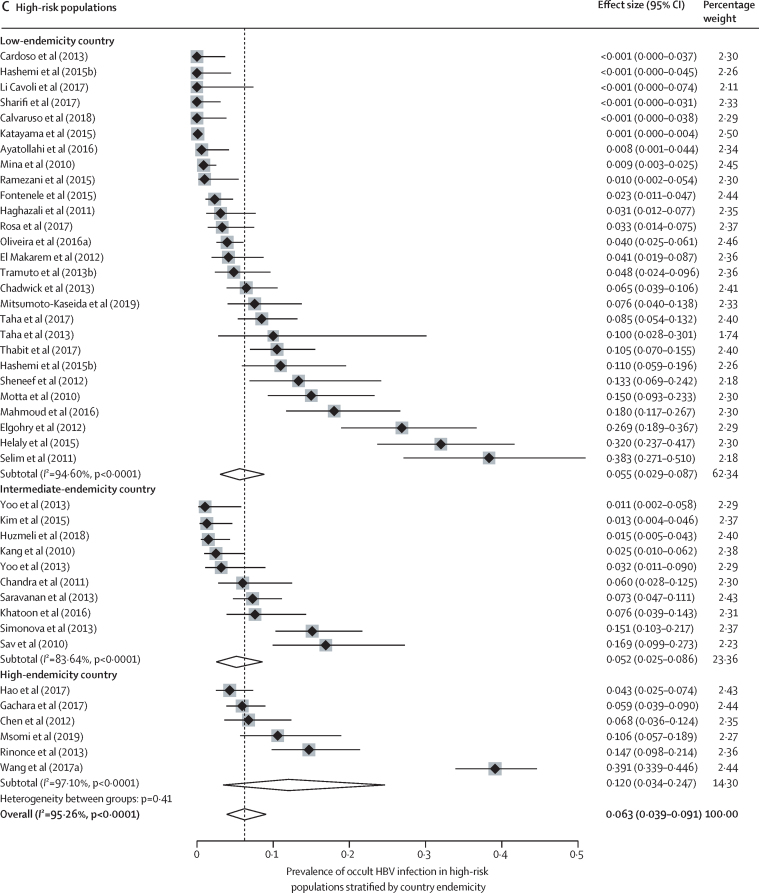

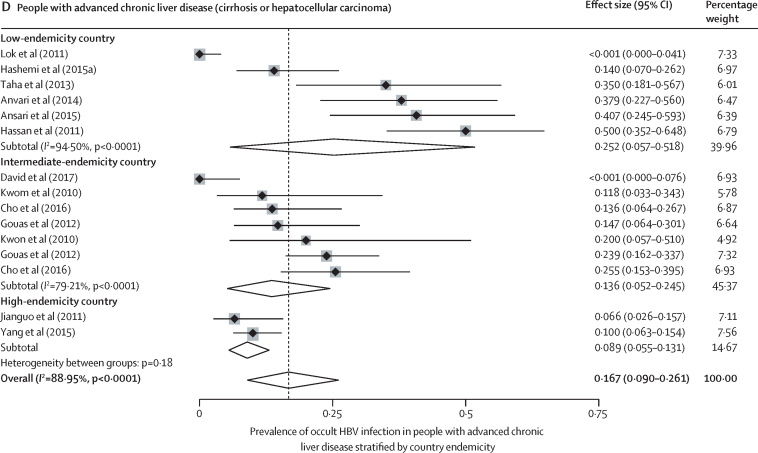
Prevalence of occult HBV infection by type of study population and country endemicity Low-risk populations include the general population, healthy health-care workers, and pregnant women; high-risk populations include patients living with HIV, patients infected with hepatitis C virus, or patients on haemodialysis. HBV=hepatitis B virus.

Occult HBV infection prevalence varied with the study population and geographical location. In blood donors, occult HBV infection prevalence mirrored HBV endemicity: 0·98% (0·44–1·72) in high-endemicity countries, 0·12% (0·04–0·23) in intermediate-endemicity countries, and 0·06% (0·00–0·26) in low-endemicity countries (*I*^2^=97·72%; p value for heterogeneity between groups was 0·0012; figure 2). In low-risk groups other than blood donors, occult HBV infection prevalence was 5·1% (2·7–8·0) in high-endemicity countries, 1·5% (0·0–5·5) in intermediate-endemicity countries, and 2·6% (0·3–6·8) in low-endemicity countries, (*I*^2^=76·37%; p=0·22; figure 2). In high-risk groups, the occult HBV infection prevalence was 12·0% (3·4–24·7) in high-endemicity countries, 5·2% (2·5–8·6) in intermediate-endemicixries, and 5·5% (2·9–8·7) in low-endemicity countries (*I*^2^=95·26%; p=0·41; figure 2). In people with advanced chronic liver disease, occult HBV infection prevalence was 8·9% (5·5–13·1) in high-endemicity countries, 13·6% (5·2–24·5) in intermediate-endemicity countries, and 25·2% (5·7–51·8) in low-endemicity countries (*I*^2^=89%; p<0·0001; figure 2). In these subgroup analyses, there was an intermediate-to-high degree of heterogeneity (range of *I*^2^ for heterogeneity within subgroups was 55–98%). The WHO region in which a study was conducted was less predictive of occult HBV infection prevalence than endemicity (appendix pp 7–8).

245 studies reported the type of HBsAg assay used: 133 (54%) used ELISA, 79 (32%) used chemiluminescent immunoassay, 27 (11%) used multiple assays, and six (2%) used radioimmunometric assays (appendix pp 17–18). None used lateral flow-based HBsAg rapid diagnostic tests. The pooled occult HBV infection prevalence, regardless of endemicity or population group, tended to increase with an increase in the limit of detection (LOD) of HBsAg assay: <0·1% (95% CI 0·0–0·0) using chemiluminescent immunoassays, 3·2% (2·9–3·5) using ELISA, and 4·4% (1·7–8·1) using radioimmunometric assays. A similar tendency persisted when restricting the analysis to blood donors (appendix p 18). However, this observed correlation was confounded by the country-level endemicity: across 14 studies on blood donors describing the type of HBsAg assay, all eight studies using chemiluminescent immunoassay were conducted in either low-endemicity (three) or intermediate-endemicity (five) countries, whereas all but one study using ELISA were conducted in resource-limited high-endemicity countries (appendix p 18). Further subgroup analyses were not possible due to small subgroups.

Regarding HBV DNA assays, all included studies used intermediate-to-high sensitivity tests for HBV DNA (LOD range 1–22 IU/mL) and we did not observe any important variation in occult HBV infection prevalence by the assay type (appendix p 19). There were insufficient data to adequately assess the effect of LOD of HBsAg and HBV DNA assays on occult HBV infection prevalence (appendix pp 20–23).

**Figure 3:**
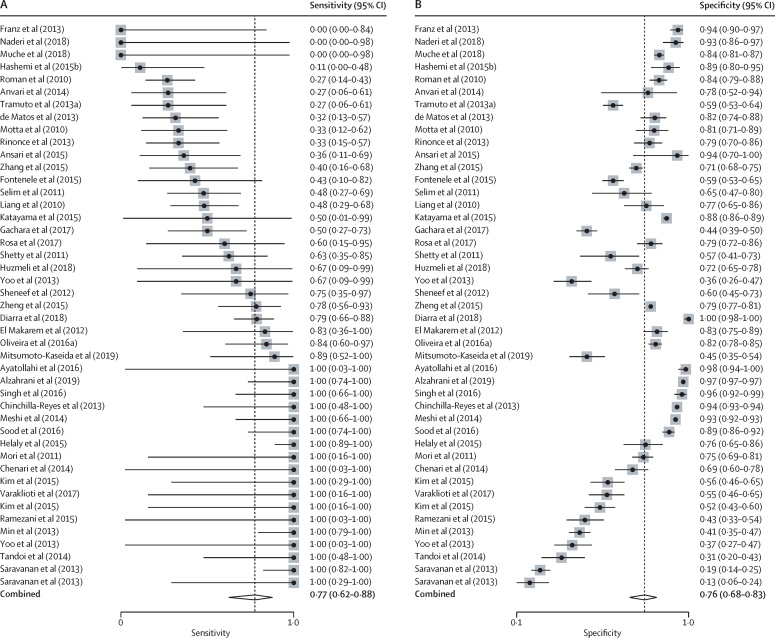
Pooled sensitivity and specificity of anti-HBc to indicate the presence of occult HBV infection HBV=hepatitis B virus.

45 studies assessed anti-HBc status in both HBsAg-negative and HBV DNA-positive people (ie, those with occult HBV infection) and HBsAg-negative and DNA-negative people (those without occult HBV infection; appendix pp 12–16). The pooled sensitivity of anti-HBc to detect the presence of occult HBV infection was 77% (95% CI 62–88) and the pooled specificity was 76% (68–83; figure 3). The area under the HSROC curve was 0·83 (95% CI 0·80–0·86; appendix p 15).

The adapted funnel plots showed no asymmetry for low-risk or high-risk populations, and people with advanced chronic liver disease, but did show asymmetry and therefore potential publication bias for blood donors (appendix pp 25–26). The risk of bias assessment mainly highlighted the scarcity of reporting for the covariates, including sex (reported by 54% of studies) and age (53%) distributions, study period (79%), and the LOD of HBsAg assays (6%) and HBV DNA assays (27%; appendix pp 27–28).

## Discussion

We performed a systematic review and meta-analysis to estimate the prevalence of occult HBV infection globally and in specific population groups, pooling results from 375 studies and 140 521 993 individuals tested for occult HBV infection. By attempting this, we identified an important knowledge gap: the global prevalence of occult HBV infection cannot be accurately estimated at present. One reason for this is the scarcity of studies targeting the general population. The second reason is the general over-representation of data from the Western Pacific, Eastern Mediterranean, and European Regions in the literature, with very few studies having been conducted in the African and South-East Asian Regions, and the Region of the Americas. Nevertheless, the existing data suggest that a non-negligible proportion of people carry occult HBV infection, particularly in high-endemicity countries and in high-risk groups worldwide. Considering that people with occult HBV infection might transmit the virus and have an increased risk of developing hepatocellular carcinoma, the high occult HBV infection prevalence probably translates into a considerable clinical and economic impact at a global scale.

We also found substantial variations of occult HBV infection prevalence according to the tissue type used, with increased rates when HBV DNA was detected in the liver rather than plasma or serum samples. However, this observation is affected by selection bias. Among the 36 studies that measured HBV DNA in 2598 liver tissues, 31 (86%) of these studies targeted patients with cryptogenic liver disease and five (14%) examined explanted donor tissue or unspecified control populations.

Recent studies have confirmed cases of transfusion-transmitted HBV due to occult HBV infection.^13^, ^24^, ^25^, ^26^ Our results suggest that in highly endemic countries (HBsAg prevalence ≥5·0%), approximately one in 100 HBsAg-negative blood donors might carry occult HBV infection and will be overlooked unless donated blood is systematically screened for HBV DNA. Because most highly endemic countries are LMICs,^17^, ^27^ sparse access to HBV DNA testing in blood banks remains a key barrier to eliminate blood-borne infections in these countries.^28^ This issue should be particularly relevant in the WHO African region, the area known to have the highest HBsAg prevalence worldwide.^17^ Our systematic review only identified 24 African studies, representing just 6% of the studies included in this meta-analysis (table), which highlights the under-representation of this region in the literature. A recent population-based study in The Gambia reported that 9·4% of the HBsAg-negative general population carry occult HBV infection and 12·9% of cases of advanced chronic liver disease were attributable to occult HBV infection in HBsAg-negative individuals,^29^ suggesting that, in high-endemicity countries, a substantial proportion of the general population might carry occult HBV infection.

In contrast to low-risk populations, high-risk groups (people living with HIV, HCV, haemodialysis, or advanced chronic liver disease) have high occult HBV infection prevalence irrespective of the country-level endemicity. This finding might support the need for HBV DNA-based screening in this population, because early identification of occult HBV infection might offer clinical benefit. An association between occult HBV infection and hepatocellular carcinoma has been frequently observed in systematic reviews of both case-control studies and prospective cohort studies.^11^, ^30^ Occult HBV infection is also a well established risk factor for HBV reactivation following immunosuppression.^31^

It is important to emphasise the intrinsic association between assay sensitivity and occult HBV infection prevalence, whereby a low-sensitivity HBsAg assay or high-sensitivity NAT will increase occult HBV infection prevalence estimations. Due to insufficient available data, we were unable to reliably separate the effects of HBsAg assay type from the effects of endemicity and specific population groups on occult HBV infection prevalence. However, recent studies suggest that newer-generation, high-sensitivity HBsAg assays successfully detect additional cases of positive HBsAg in populations formally known to be negative for HBsAg.^32^ In addition, although 138 studies included in our meta-analysis reported HBV NAT assay brand and model, we noted that often the reported assay sensitivity, if reported at all, was different to the assay sensitivity reported in the manufacturer's catalogue, and endemicity and population groups confounded analysis attempts based on LOD. Overall, it was impossible to reliably estimate the dual effect of HBsAg or NAT assay LOD on occult HBV infection prevalence, or perform an analysis with stricter inclusion criteria based on the LOD of assays. The evident heterogeneity of assay types used worldwide poses a considerable restriction to estimating the occult HBV infection prevalence. Standardisation of methodology could significantly shift our current understanding of occult HBV infection and its prevalence in the future.

Limited access to HBV DNA NAT represents a serious obstacle for the identification of occult HBV infection cases in highly endemic countries with limited resources. In this context, the use of a low-cost serological test alternative to HBV DNA NAT could provide a solution if such a test proves to be accurate. Therefore, we assessed the performance of anti-HBc to identify occult HBV infection. In our subgroup analysis, occult HBV infection prevalence was less than 0·1% (95% CI 0·0–0·0) in studies targeting HBsAg-negative people irrespective of anti-HBc serostatus, and 9·9% (7·9–12·2) in studies exclusively including HBsAg-negative people positive for anti-HBc, confirming that positive anti-HBc is an important risk factor for occult HBV infection. However, its capacity to discriminate people with occult HBV infection from people without any type of HBV infection was not sufficiently high for it to be used as a diagnostic test alternative to HBV DNA NAT: the pooled sensitivity was 77% and specificity was 76%. It would not be advisable to use anti-HBc as a sole biomarker to screen donated blood for occult HBV infection, even in a resource-limited context.

Our systematic review suggests that occult HBV infection should not be neglected as a global health issue. Corroboration of our results with the growing literature on occult HBV infection, its transmissibility, and its association with cirrhosis and hepatocellular carcinoma implies that the proportion of new cases of chronic HBV infection attributable to occult HBV infection, as well as the fraction of advanced chronic liver disease attributable to transmission from people with occult HBV infection, is unlikely to be negligible. Nevertheless, occult HBV infection has been a missing parameter from current global guidance on viral hepatitis testing,^3^ blood product safety,^33^ and HBV elimination.^34^ Apart from a single study,^33^ the population-attributable fraction for the effect of occult HBV infection has been poorly estimated. The impact of occult HBV infection on the 2030 viral hepatitis elimination targets, defined by a 90% reduction in new HBV cases and a 65% reduction in HBV-related mortality compared with the 2015 baseline, should be urgently examined.

Our systematic review has limitations. First, given the low number of studies, we could not estimate the global and regional occult HBV infection prevalence by pooling the general population estimates with inverse probability weighting using population weights. We could not include studies published after Aug 14, 2019, and an update of the literature search and analysis was not feasible during the COVID-19 pandemic. However, our main conclusions are unlikely to be substantially affected by the recency of the search. Second, we observed high degrees of heterogeneity in the prevalence estimates. The *I*^2^ statistic remained high (55–98%) even after stratifying the meta-analyses by the type of study participants and country-level endemicity. Third, we could not stratify occult HBV infection cases by viral load concentration, even though it is probable that different pathophysiological mechanisms account for highly viraemic occult HBV infection (eg, mutations in gene encoding HBsAg) on the one hand, and low or intermittent viraemia on the other.^4^ However, our research question is pragmatic rather than biological, and addresses how many people who might potentially transmit HBV to others or develop liver disease might be missed with the current screening strategy, which is based on HBsAg alone. Fourth, we restricted our systematic review to the adult population, although occult HBV infection in children has been previously reported even in children vaccinated for HBV.^35^, ^36^ Finally, we did not consider the sensitivity of HBV NAT as an inclusion criterion, and subgroup analyses based on a low LOD were not possible due to the scarcity of data reported by currently published studies.

In conclusion, occult HBV infection is common and clinically significant, but under-researched and under-recognised. Our study confirms that occult HBV infection is a particular problem in HBV-endemic areas and across high-risk groups worldwide, indicating the need for better access to occult HBV infection diagnosis. HBV DNA NAT remains expensive and inaccessible in many resource-limited, highly endemic countries. As an alternative tool, we assessed the performance of anti-HBc serology, but the pooled sensitivity and specificity were suboptimal for the purpose of diagnosing occult HBV infection. Population-based serosurveys on occult HBV infection targeting the general population and prospective studies on the mechanisms and outcomes of occult HBV infection are needed, as are modelling studies including occult HBV infection as a variable that could potentially jeopardise HBV elimination. In the meantime, occult HBV infection should no longer be overlooked in hepatitis B elimination programmes.

## Data sharing

The full search strategy and key results used to generate data that inform the conclusion of this systematic review can be found in the appendix.

## Declaration of interests

ML is a recipient of a new investigator research grant from the Medical Research Council and research grants from Gilead Sciences and Viiv Healthcare. GN has been awarded a research fellowship from Gilead Sciences and the Wellcome Trust. YS is a recipient of a research grant by Gilead Sciences and receives lecture fees from Gilead Sciences. All other authors declare no competing interests.
