# Supplementary material for: Prevalence of occult hepatitis B virus infection in adults: a systematic review and meta-analysis
Source: Lancet Gastroenterol Hepatol. 2022 Aug 10;7(10):932–42. doi: 10.1016/S2468-1253(22)00201-1 (PMC9630161; doi:10.1016/S2468-1253(22)00201-1)
Supplement: Supplementary appendix [file mmc1.pdf]

THE LANCET
Gastroenterology & Hepatology

## Supplementary appendix

This appendix formed part of the original submission and has been peer reviewed. We post it as supplied by the authors.

Supplement to: Im YR, Jagdish R, Leith D, et al. Prevalence of occult hepatitis B virus infection in adults: a systematic review and meta-analysis. *Lancet Gastroenterol Hepatol* 2022; published online Aug 9. https://doi.org/10.1016/S2468-1253(22)00201-1.

**Appendix**

**Overview**


| | |
|---|---|
| Appendix 1: Search terms | p2-3 |
| Appendix 2: Country-level endemicity | p4-6 |
| Appendix 3: OBI prevalence by World Health Organization (WHO) region | p7-8 |
| Appendix 4: Subgroup analyses | p9-11 |
| Appendix 5: OBI and anti-HBc | p12-16 |
| Appendix 6: OBI and analytical sensitivity of assays used | p17-23 |
| Appendix 7: HBV genotypes in people with OBI | p24 |
| Appendix 8: Publication bias assessment | p25-26 |
| Appendix 9: Risk of bias assessment | p27-28 |
| Appendix 10: Summary of all included studies | p29-55 |
| References in this document | p56 |

## Appendix 1: Search terms

MEDLINE(R) ALL (Ovid) [1946 to Aug 14, 2019]

1. occult.mp.
2. exp hepatitis B/ or exp hepatitis B virus/ or exp hepatitis B surface antigens/
3. (hepatitis b or hepatitis-b or ("type b" adj1 hepatitis) or hbv or hep b or hep-b or hbsag or hbs-ag or hbs antigen* or hbs-antigen* or hepatitis b surface antigen* or hepatitis-b surface antigen*).mp.
4. 2 or 3
5. 1 and 4
6. obi.mp.
7. 5 or 6
8. exp prevalence/ or exp seroepidemiologic studies/ or exp "surveys and questionnaires"/
9. (prevalen* or seroprevalen* or sero-prevalen* or seroepidemi* or sero-epidemi* or survey* or proportion* or percent* or rate* or inciden*).mp.
10. 8 or 9
11. 7 and 10

Embase Classic+Embase (Ovid) [1947 to 2019 Aug 14]

1. occult.mp
2. exp Hepatitis B/ or exp Hepatitis B Virus/ or exp Hepatitis B Surface Antigen/ or exp Hepatitis B Antigen/
3. (hepatitis b or hepatitis-b or ("type b" adj1 hepatitis) or hbv or hep b or hep-b or hbsag or hbs-ag or hbs antigen* or hbs-antigen* or hepatitis b surface antigen* or hepatitis-b surface antigen*).mp.
4. 2 or 3
5. 1 and 4
6. obi.mp.
7. 5 or 6
8. exp prevalence/ or exp seroepidemiology/ or exp seroprevalence/ or exp questionnaire/ or exp health survey/
9. (prevalen* or seroprevalen* or sero-prevalen* or seroepidemi* or sero-epidemi* or survey* or proportion* or percent* or rate* or inciden*).mp.

10. 8 or 9
11. 7 and 10

Global Health (Ovid) [Global Health 1973 to 2019 Week 33]

1. occult.mp.
2. exp hepatitis b/ or exp hepatitis b virus/ or exp surface antigens/
3. (hepatitis b or hepatitis-b or ("type b" adj1 hepatitis) or hbv or hep b or hep-b or hbsag or hbs-ag or hbs antigen* or hbs-antigen* or hepatitis b surface antigen* hepatitis-b surface antigen*).mp.
4. 2 or 3
5. 1 and 4
6. obi.af.
7. 5 or 6
8. exp serological surveys/ or exp seroprevalence/ or exp disease prevalence/ or exp surveys/ or exp questionnaires/
9. (prevalen* or seroprevalen* or sero-prevalen* or seroepidemi* or sero-epidemi* or survey* or proportion* or percent* or rate* or inciden*).mp.
10. 8 or 9
11. 7 and 10

Web of Science Core Collection [All Years 1970 - 2019]

1. TS=occult
2. TS=(hepatitis b OR hepatitis-b OR (type b NEAR/1 hepatitis) OR hbv OR hep B OR hep-b OR hbsag OR hbs-ag OR HBS antigen* OR hbs-antigen* OR hepatitis B surface antigen* or hepatitis-b surface antigen*)
3. #1 AND #2
4. TS=OBI
5. #3 OR #4
6. TS=(prevalen* or seroprevalen* or sero-prevalen* or seroepidemi* or sero-epidemi* or survey* or proportion* or percent* or rate* or inciden*)
7. #5 AND #6

## Appendix 2: Country-level endemicity

Estimates of country-level HBV endemicity were derived from POLARIS estimates of HBsAg prevalence[1] and, if these were not available, from the meta-analysis by Schweitzer *et al.*[2] For one country, Botswana, data were available from neither source, and we inferred the endemicity from neighbouring countries. Based on the HBsAg prevalence, we categorised countries into low-endemicity (HBsAg prevalence is <2.00%), intermediate-endemicity (2.00-4.99%) and high-endemicity (≥5.00%) countries in line with the WHO guidelines on testing for HBV.[3]

| Country | HBsAg prevalence | Endemicity allocated | Source used |
|---|---|---|---|
| Albania | 6.9% | High | POLARIS |
| Argentina | 0.2% | Low | POLARIS |
| Australia | 1.0% | Low | POLARIS |
| Azerbaijan | 1.8% | Low | POLARIS |
| Botswana | N/A | High | Inferred from neighbouring countries |
| Brazil | 0.4% | Low | POLARIS |
| Bulgaria | 3.2% | Intermediate | POLARIS |
| Burkina Faso | 6.1% | High | POLARIS |
| Cameroon | 6.8% | High | POLARIS |
| Canada | 0.6% | Low | POLARIS |
| China | 6.1% | High | POLARIS |
| Cote d'Ivoire | 8.9% | High | POLARIS |
| Cuba | 0.6% | Low | POLARIS |
| Egypt | 1.0% | Low | POLARIS |
| France | 0.5% | Low | POLARIS |
| Gambia | 4.8% | Intermediate | POLARIS |
| Germany | 0.3% | Low | POLARIS |
| Greece | 1.8% | Low | POLARIS |
| Hong Kong | 6.4% | High | POLARIS |
| India | 2.5% | Intermediate | POLARIS |

| | | | |
|---|---|---|---|
| Indonesia | 6.8% | High | POLARIS |
| Iran | 1.7% | Low | POLARIS |
| Iraq | 3.5% | Intermediate | POLARIS |
| Italy | 0.6% | Low | POLARIS |
| Japan | 0.6% | Low | POLARIS |
| Jordan | 2.4% | Intermediate | POLARIS |
| Kazakhstan | 2.7% | Intermediate | POLARIS |
| Kenya | 1.2% | Low | POLARIS |
| Lebanon | 1.2% | High | POLARIS |
| Libya | 1.5% | Low | POLARIS |
| Malaysia | 0.9% | Low | POLARIS |
| Mexico | 0.1% | Low | POLARIS |
| Mongolia | 4.1% | Intermediate | POLARIS |
| Mozambique | 7.5% | High | POLARIS |
| Netherlands | 0.3% | Low | POLARIS |
| Nigeria | 11.2% | High | POLARIS |
| Pakistan | 2.1% | Intermediate | POLARIS |
| Philippines | 9.8% | High | POLARIS |
| Poland | 0.9% | Low | POLARIS |
| Portugal | 1.2% | Low | POLARIS |
| Romania | 3.4% | Intermediate | POLARIS |
| Slovakia | 1.6% | Low | POLARIS |
| South Africa | 6.7% | High | Schweitzer et al. |
| South Korea | 2.4% | Intermediate | POLARIS |
| Spain | 0.6% | Low | POLARIS |
| Sudan | 5.3% | High | POLARIS |
| Switzerland | 0.5% | Low | POLARIS |
| Syria | 5.7% | High | POLARIS |
| Taiwan | 9.4% | High | POLARIS |
| Thailand | 3.5% | Intermediate | POLARIS |
| Tunisia | 3.9% | Intermediate | POLARIS |
| Turkey | 2.6% | Intermediate | POLARIS |

| | | | |
|---|---|---|---|
| Ukraine | 1.5% | Low | Schweitzer et al. |
| United Arab Emirates/Saudi Arabia | 1.6% | Low | POLARIS |
| United Kingdom | 0.7% | Low | POLARIS |
| USA | 0.3% | Low | POLARIS |
| Vietnam | 8.2% | High | POLARIS |
| Yemen | 3.2% | Intermediate | POLARIS |

## Appendix 3: OBI prevalence by World Health Organization (WHO) region

**Appendix 3, Table 1:** OBI prevalence stratified by WHO region and by population type.

| | **Blood donors** | **Low-risk groups other than blood donors** | General population | Healthcare Workers | Pregnant women | **High-risk groups** | HIV | HCV | Haemodialysis | **People with advanced liver disease** | HCC | Cirrhosis |
|---|---|---|---|---|---|---|---|---|---|---|---|---|
| **AFR** | | | 7.4% (5.0-10.8) n=323 (1 study) | | | 12.7% (6.7-20.2) n=1,087 (6) $I^2$= | | | | | | |
| **AMR** | <0.01% (0.00-0.01) N=70,225 (2) Median=0.01 % | 5.5% N=1,007 (1) | 5.5% N=1,007 (1) | N=0 | N=0 | 5.0% (2.0-9.2) N=1,032 (4) $I^2$=84% | 4.0% N=480 (1) | 3.3% N=151 (1) | 4.4% (2.6-6.7) N=401 (2) Median=8.7 % | <0.1% N=91 (1) | <0.1 % N=91 (1) | N=0 |
| **EMR** | 0.08% (0.00-0.25) N=84,962 (5) $I^2$=92% | 1.4% (0.1-3.8) N=200 (2) Median=3.3 % | <0.1% N=80 (1) | 3.3% N=120 (1) | N=0 | 8.8% (4.3-14.5) N=1,722 (16) $I^2$=92% | N=0 | 11.0% (4.8-19.3) N=865 (8) $I^2$=96% | 6.8% (1.3-15.5) N=857 (8) $I^2$=94% | 34.4% (17.6-53.4) N=146 (4) $I^2$=81% | 50.0 % N=40 (1) | 29.2% (12.2-49.6) N=106 (3) Median=37.9 % |
| **EUR** | N=0 | <0.1% N=91 (1) | N=0 | <0.1% N=91 (1) | N=0 | 3.3% (0.7-7.2) N=1,374 (9) $I^2$=90% | 5.8% (3.6-8.5) N=361 (2) Median=5.7 % | 2.4% (0.0-15.9) N=347 (3) Median=7.6 % | 2.7% (0.0-8.8) N=665 (4) $I^2$=89% | N=0 | N=0 | N=0 |
| **SEAR** | 0.20% (0.06-0.41) N=159,512 (5) $I^2$=98% | 1.7% (0.1-5.8) N=376 (4) $I^2$=72% | 4.9% (2.4-8.0) N=243 (2) Median=4.6 % | <0.1% (0.0-1.2) N=133 (2) Median=0.0 % | N=0 | 9.0% (4.8 -14.4) N=503 (3) Median=7.3 % | 6.0% N=100 (1) | 7.3% N=260 (1) | 14.7% N=143 (1) | <0.1% N=47 (1) | <0.1 % N=47 (1) | N=0 |
| **WPR** | 0.22% (0.11-0.36) N=288,952 (8) $I^2$=96% | 3.3% N=121 (1) | 3.3% N=121 (1) | N=0 | N=0 | 5.0% (0.3-14.2) N=3,162 (9) $I^2$=98% | 7.6% N=119 (1) | 1.9% (0.2-5.0) N=596 (2) Median=21.7 % | 1.9% (0.2-5.0) N=2,447 (6) $I^2$=89% | 12.4% (7.1-18.7) N=349 (6) $I^2$=47% | 9.2% (5.8-13.1) N=285 (4) $I^2$=0% | 21.4% (11.8-32.7) N=64 (2) Median=18.7 % |

Effect estimates are stated as pooled prevalence (95% confidence interval), N = number of individuals tested (number of studies in brackets), $I^2$ = heterogeneity statistic. Abbreviations: AFR = African region, AMR = Region of the Americas, EMR = Eastern Mediterranean Region, EUR = European Region, SEAR = South-East Asia Region, WPR = Western Pacific Region, HCC = hepatocellular carcinoma, HCV = hepatitis C virus, HIV = human immunodeficiency virus.

There are fewer studies than originally included (Table 1) as we performed subgroup analyses and only included blood samples, pre-specified population groups and serological criteria that allow pooling studies.

## Appendix 4: Subgroup analyses

OBI prevalence was predicted to differ according to the following definitions of study outcomes:

1. Tissue in which HBV DNA is detected (liver, serum/plasma, peripheral blood mononuclear cells [PBMCs]) due to an inflated prevalence in studies on liver[4] and PBMCs[5] in line with previous reports. The results are summarised in the 'Results' section of the main manuscript as well as Appendix 4, Table 1.

2. Serological criteria used by study to test for OBI (all HBsAg- regardless of anti-HBc and anti-HBs status, HBsAg- anti-HBc+ regardless of anti-HBs status, HBsAg- anti-HBc+ anti-HBs-, HBsAg- anti-HBc- anti-HBs-, any other criteria) due to previous reports of higher OBI prevalence in anti-HBc-positive individuals.[6] The results are summarised below in Appendix 4, Table 1.

**Appendix 4, Table 1:** OBI prevalence according to serological criteria to test for OBI.

| | Plasma/Serum | PBMCs | Liver |
|---|---|---|---|
| HBsAg - | <0.1% (95%CI 0.0-0.0) N=139,938,377 individuals (214 studies) $I^2$=100% | 2.1% (95%CI 0.0-10.1) N=1,106 individuals (3 studies) $I^2$=96% | 34.3% (95%CI 25.9-43.3) N=2,290 individuals (33 studies) $I^2$=95% |
| HBsAg - anti-HBc + | 9.9% (7.9-12.2) N=188,420 (82) $I^2$=98% | N=0 | 36.3% (29.3-43.6) N=178 (2) Median=36.5% |
| HBsAg - anti-HBc + anti-HBs - (isolated anti-HBc) | 11.9% (7.3-17.4) N=15,249 (25) $I^2$=98% | N=0 | 42.3% N=130 (1) |

| | | | |
|---|---|---|---|
| HBsAg -<br>anti-HBc-<br>anti-HBs- | 7.4% (1.0-18.3)<br>N=369,866 (5)<br>$I^2$=98% | N=0 | N=0 |
| HBsAg- and any other criteria | 9.8% (3.2-19.3)<br>N=6,377 (10)<br>$I^2$=98% | N=0 | N=0 |

Subgroup analyses were also performed to investigate the high heterogeneity observed in our results, both based on different study population criteria and pre-specified sources of heterogeneity. These included:

3. HBV endemicity of country in which the study was conducted (country endemicity), as summarised in the 'Methods' and 'Results' of the main manuscript.
4. Geographical location (WHO region), as summarised below in Appendix 4, Table 1.
5. Whether studies included first-time donors only, or also repeat donors, as summarised below in Appendix 4, Table 2.

We observed a significantly higher OBI prevalence in studies that test for HBV DNA in liver tissue rather than serum/plasma samples (p<0.001) (Appendix 4, Table 1). We also observed a significantly higher OBI prevalence in studies that test HBsAg-negative, anti-HBc-positive populations compared to those testing HBsAg-negative individuals irrespective of hepatitis B antibody status (p<0.001) (Appendix 4, Table 1). Finally, there was a significantly different OBI prevalence rates in studies including repeat donors compared to those on first-time donors only (p<0.001) (Appendix 4, Table 2). This did not seem to be significantly confounded by endemicity (Appendix 4, Table 2).

Therefore, for the purposes of comparing prevalence rates across different regions (by WHO region and country-level endemicity) and across different population groups, we only included studies focussing on HBsAg-negative individuals irrespective of anti-HBc status, focussed on serum/plasma samples (Figure 2 and Appendix 4, Table 1), and excluded repeat donors from the analysis.

**Appendix 4, Table 2:** OBI prevalence in studies including first-time donors only, vs. studies also including repeat blood donors or donation samples.

| First-time donors | Repeat donors |
|---|---|
| 1.1% (95%CI 0.8-1.4)<br>N=609,832 individuals<br>(44 studies)<br>$I^2$=98%<br><br>32 in high,<br>11 in intermediate,<br>23 in low-endemicity countries. | <0.1% (0.0-0.0)<br>N= 139,867,719 individuals<br>(89 studies)<br>$I^2$=100%<br><br>7 in high,<br>2 in intermediate,<br>9 in low-endemicity countries. |

## Appendix 5: OBI and anti-HBc

Figures 1 and 2 present the forest plots of OBI prevalence in blood donors positive for anti-HBc and blood donors negative for anti-HBc, respectively.

Figure 3 presents the hierarchical summary receiver operating characteristics (HSROC) curve based on logit estimates of sensitivity, specificity, and respective variances that were derived from the bivariate random-effects model using the “midas” command of Stata.[7] The HSROC curve is displayed along with the observed study data. The dashed line around the summary point estimate indicates the 95% confidence region. The area under the curve (AUC) was used as a global measure of test performance.[7]

Figure 4 presents the Chi-squared probability plot of squared Mahalanobis distances for the assessment of the bivariate normality assumption.

**Appendix 5, Figure 1:** OBI prevalence in blood donors positive for anti-HBc, stratified by endemicity. ES = effect size (OBI prevalence).

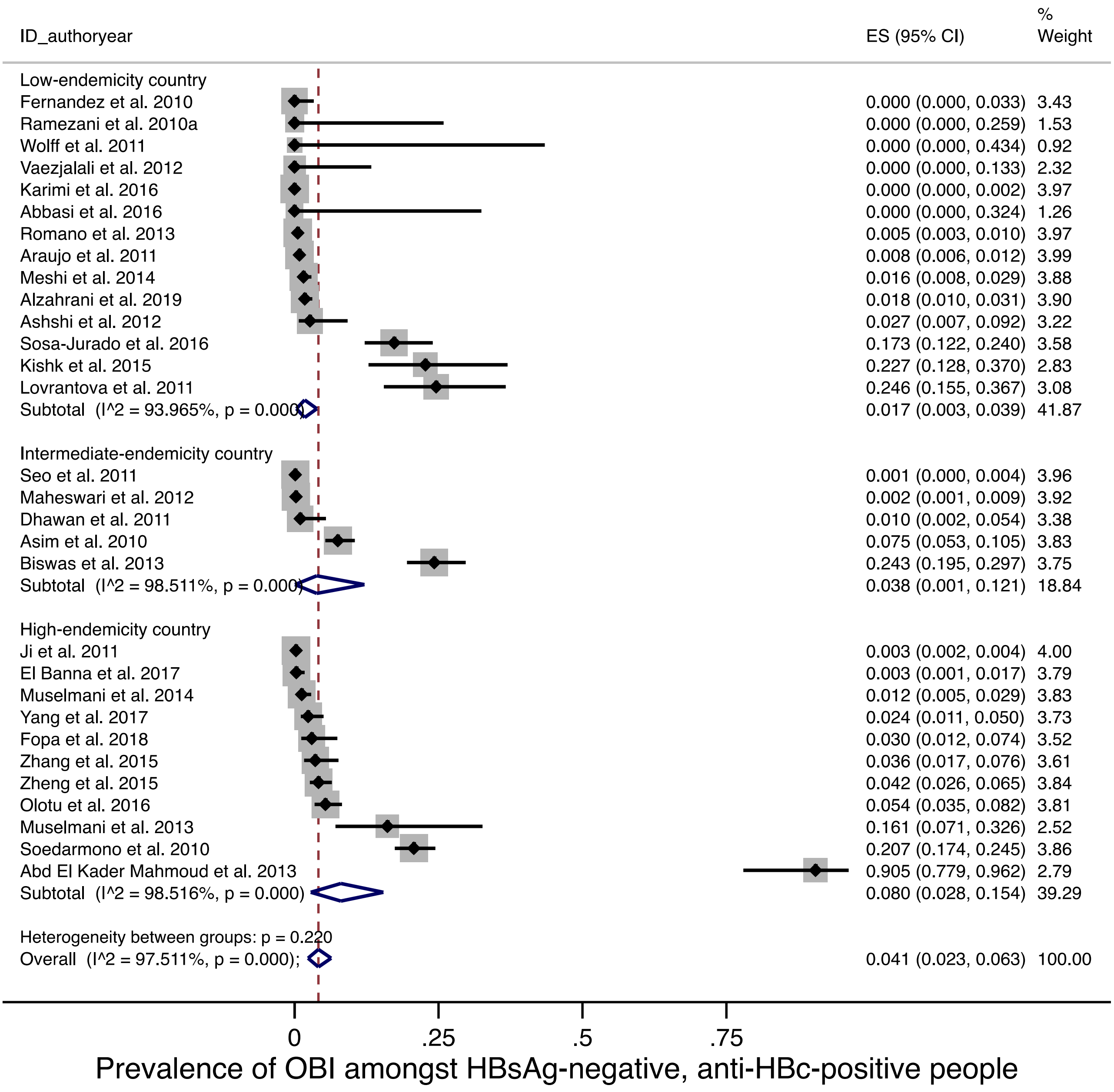

**Appendix 5, Figure 2:** OBI prevalence in blood donors negative for anti-HBc, stratified by endemicity. ES = effect size (OBI prevalence).

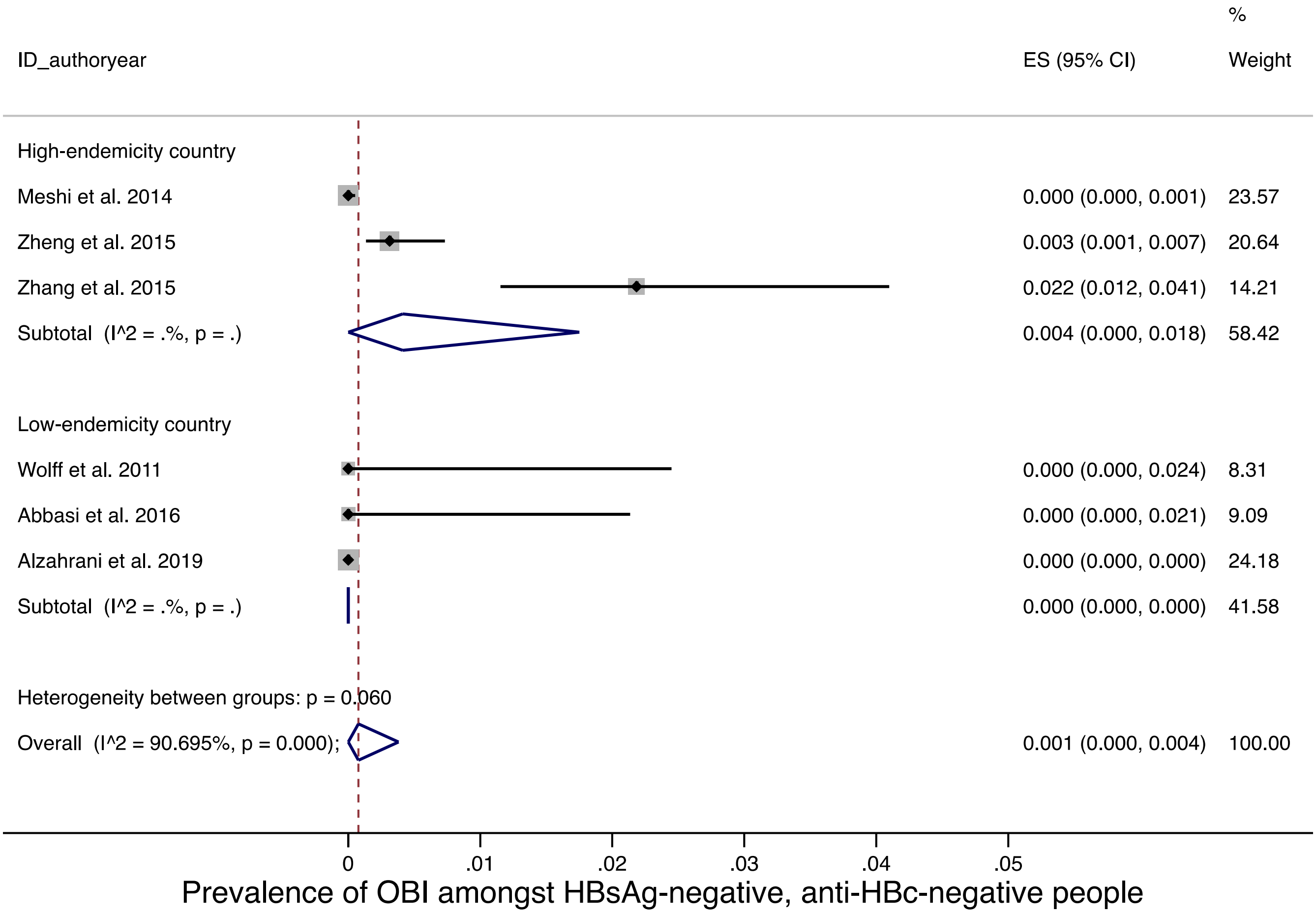

**Appendix 5, Figure 3:** Hierarchical summary receiver operating characteristic (HSROC) curve for the performance of anti-HBc to identify OBI.

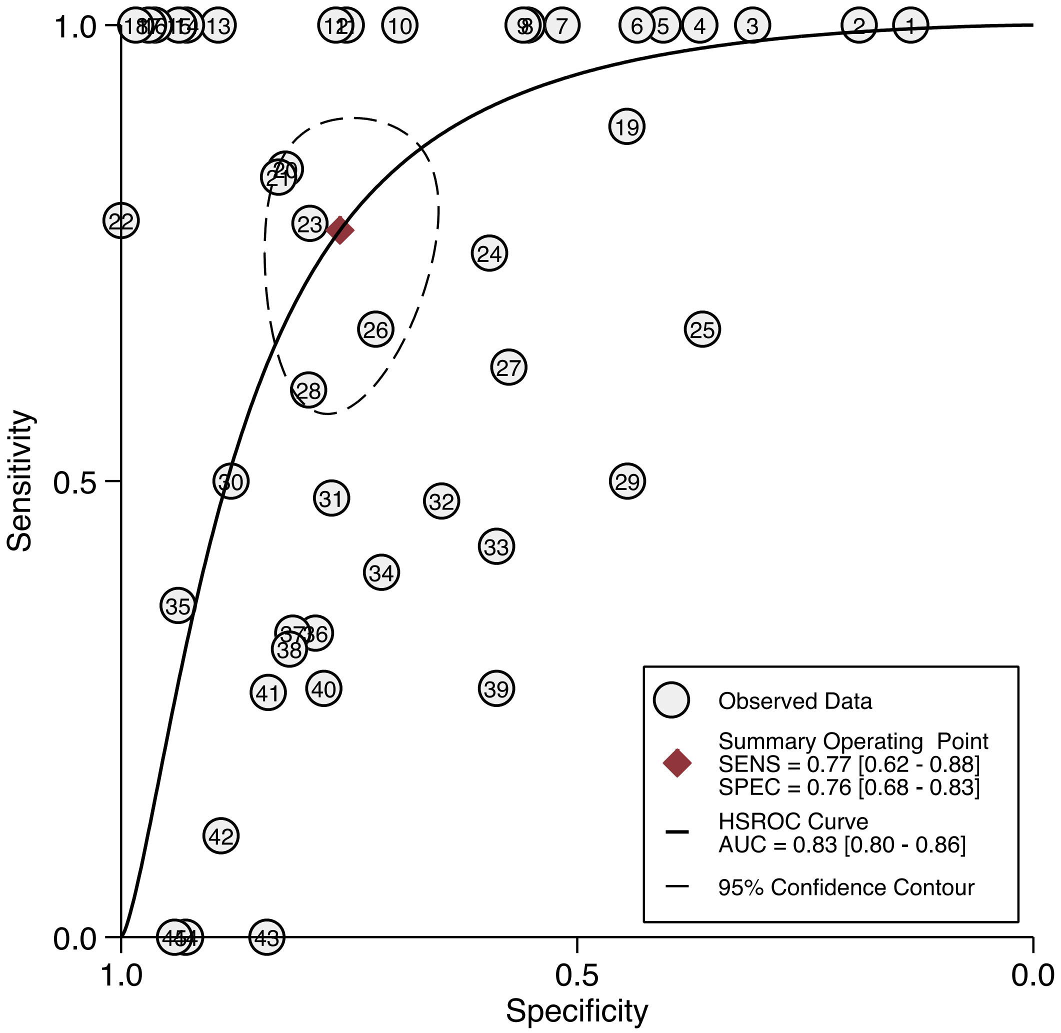


AUC = area under the curve, SENS = sensitivity, SPEC = specificity, HSROC = hierarchical summary receiver operating characteristic. Each numbered data point refers to an individual study. 95% confidence intervals are stated in square brackets. The dashed line presents the 95% confidence region.

**Appendix 6, Figure 4:** Chi-squared probability plot of squared Mahalanobis distances for assessment of the bivariate normality assumption.

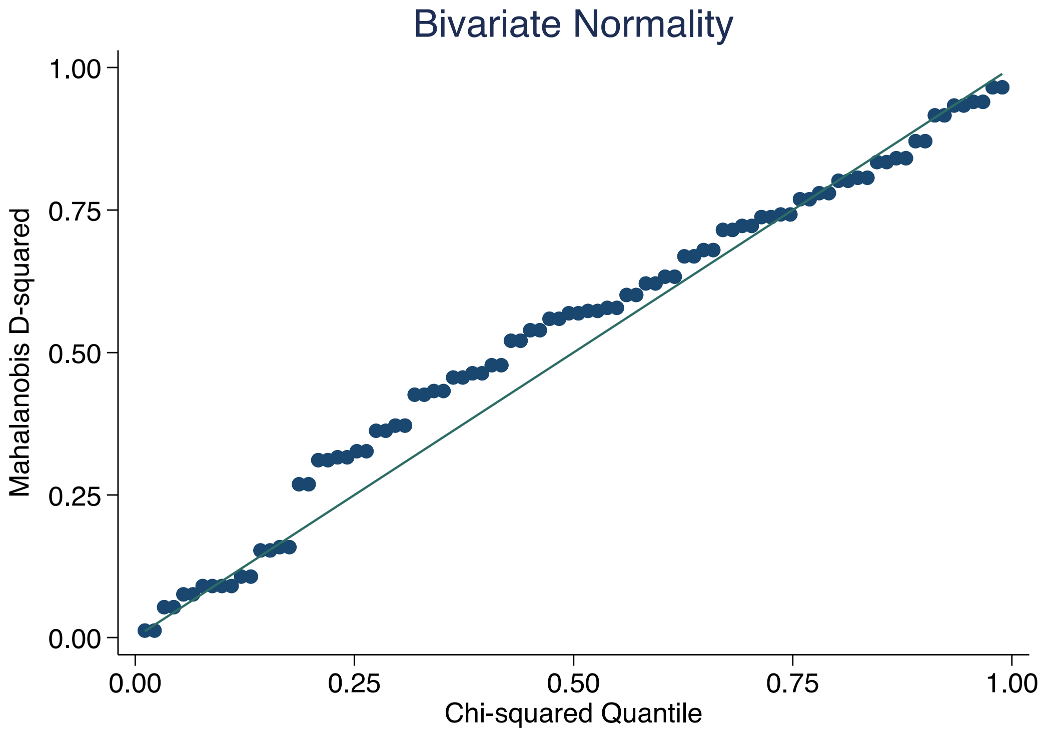

## Appendix 6: OBI prevalence and analytical sensitivity of assays used

We categorised the HBsAg assays in chemiluminescent enzyme immunoassays (CLIA), enzyme-linked immunoassays (ELISA), radioimmunometric assays (RIA), and other/unspecified assay type. In total, 245/375 studies stated the type of HBsAg assay used. Of these, 79/245 used CLIA, 133/245 used ELISA/ELIA, 6/245 used RIA, and the remaining 27/245 used multiple assays or assays of a type that was not specified. 159/275 studies also stated the exact name of the model used. In total, 46 unique HBsAg assays were used, which may have been an additional source of high heterogeneity.

The assays most commonly used across all studies are listed in Appendix 6, Table 1. The assays most commonly used in blood donor studies were PRISM (Abbott) (n=15), Architect (Abbott) (n=10) and Murex (Abbott) (n=3). Note that no study on blood donors used radioimmunometric or other previously used assays with lower sensitivity.[3]

When comparing OBI prevalence by HBsAg assay type, OBI prevalence appeared significantly higher in studies using ELIA/ELISA, both when all studies were considered ($p<0.001$) and HBsAg-negative blood donors only ($p<0.001$). OBI prevalence appeared to be highest with the use of RIA, which are generally less sensitive; however, it is difficult to draw this conclusion given that only six studies assessed this, most of them (5 out of 6 studies) in the Western Pacific Region and on high-risk populations (5 out of 6). Overall, this analysis was likely confounded by endemicity (see annotation in Appendix 6, Table 2), and further subgroup analyses were not possible due to small sample sizes.

**Appendix 6, Table 1:** The five most frequently used HBsAg assays.

| HBsAg assay brand & model | Assay type | Number of studies (out of a total of 159) |
|---|---|---|
| Architect (Abbott) | CLIA | 34 (21%) |
| PRISM (Abbott) | CLIA | 16 (10%) |
| Hepanostika (Biomerieux) | ELIA/ELISA | 9 (6%) |

| | | |
|---|---|---|
| AxSYM (Abbott) | ELIA/ELISA | 7 (4%) |
| Murex (Abbott) | ELIA/ELISA | 5 (3%) |

**Appendix 6, Table 2:** OBI prevalence according to type of HBsAg assay used.

| | **CLIA** | **ELIA/ELISA** | **RIA** |
|---|---|---|---|
| **All studies** | <0.1% (0.0-0.0)<br>$I^2$=100%<br>N= 77,046,907 (79) | 3.2% (2.9-3.5)<br>$I^2$=99%<br>N=2,279,152 (133) | 4.4% (1.7-8.1)<br>$I^2$=83%<br>N=1,569 (2) |
| **Studies on blood donors including all HBsAg- individuals** | 0.1% (0.0-0.1)<br>$I^2$=97%<br>N= 313,103 (8)<br><br>0 studies in high, 5 studies in intermediate, 3 studies in low-endemicity countries. | 1.9% (0.5-4.0%)<br>$I^2$=98%<br>N= 19,041 (6)<br><br>5 studies in high, 1 study in intermediate, 0 studies in low-endemicity countries. | N=0 |

In terms of HBV nucleic acid testing (NAT), overall, 67 different assays were used across all included studies. We categorised the assays in nested PCR, real-time PCR (RT-PCR), and other types of NAT. 304 studies specified the type of NAT used. 79/304 used nested PCR, 194/304 used RT-PCR, 31/304 used a mixture of models or unspecified models. The most commonly used models are listed in Appendix 6, Table 3.

When considering all studies, OBI prevalence appeared to be significantly higher in studies using nested PCR ($p<0.001$) (Appendix 6, Table 4). However, when restricting the analysis to blood donors and high-endemicity countries (insufficient data were available to compare low or intermediate-endemicity countries), the 95% confidence intervals overlapped and there was no increased OBI prevalence with nested PCR (Appendix 6, Table 4).

**Appendix 6, Table 3:** The five most commonly used HBV NAT assays. RT-PCR = real-time polymerase chain reaction, TMA = transcription-mediated amplification.

| HBV NAT assay brand & model | Assay type | Number of studies (out of a total of 205) |
|---|---|---|
| Nested PCR (in-house) | Nested PCR | 79 |
| Roche Cobas TaqMan | RT-PCR | 21 |
| ProCleix Ultrio | TMA | 19 |
| Applied Biosystems TaqMan | RT-PCR | 10 |
| Qiagen Artus | RT-PCR | 9 |

**Appendix 6, Table 4:** OBI prevalence according to type of HBsAg assay used.

| | Nested PCR | Real-time PCR |
|---|---|---|
| **All studies** | 12.92% (11.52-14.38)<br>$I^2$=99%<br>n=368,940 (79 studies) | <0.01% (0.00-0.01)<br>$I^2$=99%<br>n=66,086,175 (194 studies) |
| **Studies on blood donors including all HBsAg-individuals** | 1.47% (0.33-3.33)<br>$I^2$=98%<br>n=9,159 (6 studies)<br><br>5 in high,<br>1 in low-endemicity countries. | 0.03% (0.02-0.04)<br>$I^2$=99%<br>n=66,043,725 (40 studies)<br><br>13 in high,<br>10 in intermediate,<br>17 in low-endemicity countries. |
| **Studies on blood donors including all HBsAg-individuals in high-endemicity countries only** | 1.90% (0.50-4.14)<br>$I^2$=98%<br>n=18,556 (6 studies) | 0.61% (0.41-0.85)<br>$I^2$=100%<br>n=1,108,949 (13 studies) |

Next, we aimed to assess the effect of analytical sensitivity (or lower limit of detection [LOD]) of the HBsAg and HBV DNA assays used on OBI prevalence. When designing the study quality assessment (Appendix 9), we pre-defined cut-offs for low and high sensitivity assays based on current WHO guidance on testing HBV.[3] We therefore categorised HBsAg and HBV DNA assays used by at least three different studies in low-sensitivity (HBsAg LOD ≥0.13 IU/ml, HBV DNA LOD >15 IU/ml) or high-sensitivity assays (HBsAg LOD <0.13 IU/ml, HBV DNA LOD ≤15 IU/ml). Because different studies reported different analytical sensitivities for a given assay, we searched the manufacturer's catalogues and otherwise comparative analyses in the literature to settle discrepancies in reported LOD values.[8–10]

We then cross-tabulated OBI prevalence according to HBsAg LOD and HBV DNA LOD. We hypothesised that the likelihood of being diagnosed as OBI to be B>A, B>D, A>C, D>C, if:

| | HBsAg LOD <0.05 IU/ml | HBsAg LOD ≥0.05 IU/ml |
|---|---|---|
| HBV PCR LOD ≤15 IU/ml) | A | B |
| HBV PCR LOD >15 IU/ml | C | D |

The results are shown in Appendix 6, Table 5 for all blood donors, and in Appendix 6, Table 6 for all studies on serum/plasma samples for the reasons discussed below.

**Appendix 6, Table 5:** Cross-tabulation of OBI prevalence according to the sensitivity of the HBsAg assay and the sensitivity of HBV NAT across <u>blood donor</u> studies reporting these data.

| | HBsAg LOD <0.05 IU/ml | HBsAg LOD ≥0.05 IU/ml |
|---|---|---|
| HBV PCR LOD ≤15 IU/ml) | 0.022% (0.016-0.030)<br>$I^2$=99%<br>n=38,384,631 (18 studies)<br><br>3 in high,<br>3 in intermediate, | 0.008% (0.000-0.024)<br>Median=0.153%<br>n=35,608 (2 studies)<br><br>1 in low, |

| | | |
|---|---|---|
| | 11 in low-endemicity countries.<br><br>This includes studies across EUR (n=7), WPR (n=5), EMR (n=2), SEAR (n=2), AMR (n=1), mixed (n=1), and none in AFR. | 1 in intermediate-endemicity countries.<br><br>Both in EUR (n=2). |
| HBV PCR LOD >15 IU/ml | 0.006% (0.002-0.015)<br>n=70,067 (1 single study)<br><br>1 in low-endemicity countries.<br>Single study in AMR. | n=0 (no studies) |

In blood donors, using a more sensitive HBsAg assay (LOD <0.05 IU/ml) was not associated with a significantly higher OBI prevalence amongst those studies also using highly sensitive NAT (p=0.425). Insufficient data did not permit this analysis on studies using less sensitive NAT.

In terms of HBV NAT, we did observe a trend towards increased OBI detection when a higher-sensitivity assay was used (p=0.002) but this was based on a single study in the less sensitive HBV DNA assay arm, not allowing a meaningful statistical interpretation.

Because so few studies were available to perform this analysis, we also checked the same analysis including all studies on serum/plasma samples that tested HBsAg-negative individuals.

**Appendix 6, Table 5:** Cross-tabulation of OBI prevalence according to the sensitivity of the HBsAg assay and the sensitivity of HBV NAT across all studies reporting these data.

| | HBsAg LOD <0.05 IU/ml | HBsAg LOD ≥0.05 IU/ml |
|---|---|---|
| HBV PCR LOD ≤15 IU/ml) | <0.01% (0.00-0.00)<br>$I^2$=99%<br>N=38,393,625 (22)<br><br>3 in high,<br>6 in intermediate,<br>12 in low-endemicity countries. | 1.88% (3.52-7.38)<br>$I^2$=95%<br>N=35,788 (7)<br><br>1 in high,<br>6 in intermediate,<br>7 in low-endemicity countries. |
| HBV PCR LOD >15 IU/ml | 0.78% (0.03-2.23)<br>$I^2$=94%<br>N=72,578 (8)<br><br>1 in high,<br>1 in intermediate,<br>6 in low-endemicity countries.<br><br>These studies were based in the Eastern Mediterranean Region (n=4), European Region (n=3) and Region of the Americas (n=1). | 4.65% (3.38-6.10)<br>$I^2$=29%<br>N=1,478 (6)<br><br>5 in high,<br>1 in low-endemicity countries.<br><br>Half of these studies were from the African Region (n=3) and the remainder from the Western Pacific Region (n=2) and the Regions of the America (n=1). |

HBsAg assay sensitivity appeared to be predictive of OBI prevalence when pooling all studies, whereas we did not find what we expected for HBV DNA sensitivity. However, the whole analysis was confounded by the effect of country-level HBV endemicity on the association between assay sensitivity and OBI prevalence: high-endemicity countries had higher OBI prevalence and tended to use less sensitive NAT. Of 29 studies using high-sensitivity HBV DNA assays, 19 were conducted in low-endemicity countries (4 in Iran; 2 in Netherlands, Poland, Spain, and Malaysia each; 1 in Argentina, Australia, Greece, Italy, the

UEA, and USA each) and three studies were conducted in high-endemicity countries (2 in Hong Kong, 1 in Albania). Of 14 studies using low-sensitivity HBV DNA assays, 7 were conducted in low-endemic countries (2 in Germany and Italy each, 1 in Argentina, Brazil and Japan each) and 6 were conducted in high-endemic countries (2 in Botswana and China each, 1 in Nigeria and Cameroon each).

To conclude, because of this confounding, our data did not allow us to comment on the effect of analytical sensitivity of assays used on OBI prevalence conclusively. This was likely due to a combination of (i) high degrees of heterogeneity in methodology (i.e. use of a large number of different assays), (ii) the lack of reporting of methodology, (iii) important moderators of OBI prevalence identified in this study restricting the sample size of studies that could be assessed, (iv) the use of higher-sensitivity assays in higher-income countries.

## Appendix 7: HBV genotypes amongst people with OBI

## Appendix 7, Table 1

| Genotype | AFR | AMR | EMR | EUR | SEAR | WPR |
|---|---|---|---|---|---|---|
| **A** | 12 (57%) | 66 (27%) | 3 (2%) | 1 (1%) | 8 (3%) | 5 (1%) |
| **B** | 0 | 7 (3%) | 12 (8%) | 1 (1%) | 13 (5%) | 247 (42%) |
| **C** | 0 | 6 (2%) | 6 (4%) | 0 | 13 (5%) | 320 (54%) |
| **D** | 0 | 36 (15%) | 128 (83%) | 74 (93%) | 213 (86%) | 18 (3%) |
| **E** | 9 (43%) | 0 | 0 | 4 (5%) | 0 | 0 |
| **F** | 0 | 10 (4%) | 0 | 0 | 0 | 0 |
| **G** | 0 | 1 | 0 | 0 | 0 | 0 |
| **H** | 0 | 117 (48%) | 0 | 0 | 0 | 0 |
| **Mixed** | 0 | 0 | 5 (3%) | 0 | 0 | 0 |
| **Total OBI cases** | 21 | 243 | 154 | 80 | 248 | 590 |

## Appendix 8: Publication bias assessment

Please see overleaf for Appendix 8 Figure 1.

While the funnel plots were grossly symmetrical for low-risk, high-risk, and ACLD populations, they were asymmetrical for blood donors.

Although we cannot preclude a possibility that publication bias has distorted findings, we also suspect that this asymmetry may be explained by endemicity and how resourceful the country of study is. Resource-rich countries tend to conduct larger-scale studies and have lower HBV endemicity rates; therefore, larger-scale blood donor studies may observe lower OBI prevalence rates. By contrast, resource-limited countries are often unable to conduct very large studies, while also observing higher HBV endemicity and OBI prevalence rates.

**Appendix 8, Figure 1:** Funnel plot using study size including pooled population groups.

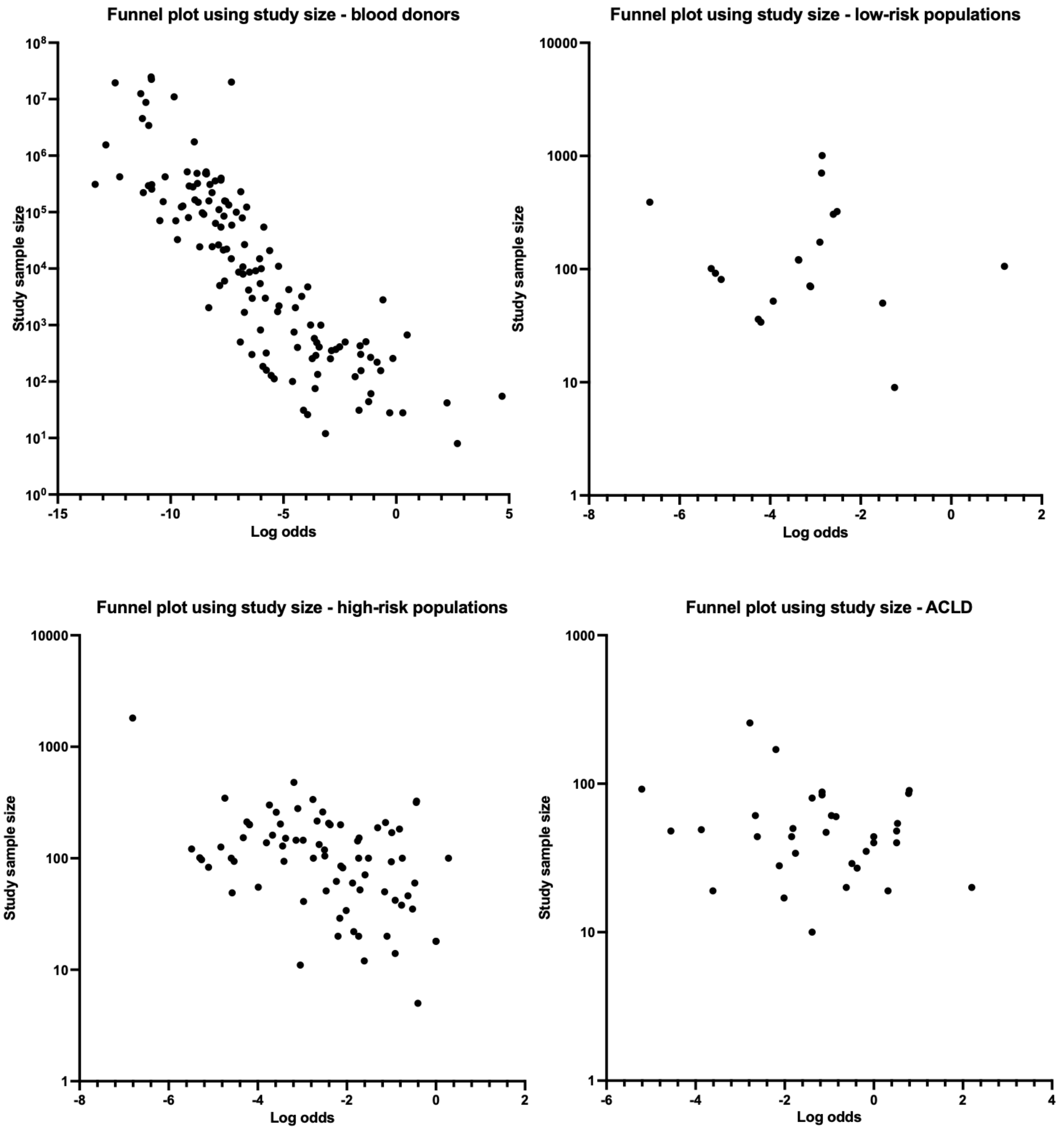

## Appendix 9: Risk of bias assessment

| Question | Number of studies that fulfil this criterion (n=375) | % | Comment |
|---|---|---|---|
| Is this either a population based or a facility-based study? If either is known, put 1. If neither, 0. | 369 | 98% | |
| Were study participants sampled consecutively? If unknown (as for most papers), put 0. | 131 | 35% | Not scored if NA. It is difficult to comment on this as most studies did not describe this and were scored 0. |
| Is the sample size >500?. | 156 | 42% | This applies to the total population size, not only to the number of people tested for OBI. Note that only 108 out of 375 (29%) studies tested >500 people for OBI. |
| Is sex distribution reported? | 202 | 54% | Not scored if NA. It is difficult to comment on this as most studies did not describe this. Some were not included in Table 1 as they declared data in a format which could not be summarised (e.g. range or proportion only). |
| Is age distribution reported? | 198 | 53% | Not scored if NA. It is difficult to comment on this as most studies did not describe this. Some were not included in Table 1 as they declared data in a format which could not be summarised (e.g. range or proportion only). |
| Is the population character (Blood donors, HIV, HCV etc) described? | 365 | 97% | |
| Is the centre where the research was conducted described? | 310 | 83% | |
| Is the country described? | 367 | 98% | |
| Is the study period defined? | 298 | 79% | This was often not the case, especially for conference abstracts. |
| Is the subject/setting appropriate? | 159 | 42% | This only scored if each of the previous three questions scored. |

| | | | |
|---|---|---|---|
| Coverage for HBsAg test (n) out of total study population (N) >=80% | 358 | 95% | |
| Coverage for HBV DNA (m) in HBsAg-negative subjects (M) >=80% | 269 | 72% | Many studies did not state why they picked a random subsample, which could have been biased. These studies emerged as outliers in our analysis and therefore we removed them from comparative/sub-group analyses. Others declared legitimate reasons, e.g. not having sufficient samples for retrospective NAT. We have included these studies and the number stated here refers to the remaining studies, of which 72% tested >=80% of eligible individuals and 28% tested <80% while giving a legitimate reason for this. |
| Is the data analysis appropriate? | 259 | 69% | This only scored if each of the previous two questions scored. |
| HBV DNA LOD <15 IU/mL (<75 copies/mL) | 103 | 27% | Not scored if NA. It is difficult to comment on this as most studies did not describe this. Also only scored if declared by study authors. |
| HBsAg <0.13 IU/mL | 22 | 6% | Not scored if NA. It is difficult to comment on this as most studies did not describe this. Also only scored if declared by study authors. |
| Is the Methodology appropriate? | 11 | 3% | This only scored if each of the previous two questions scored, therefore very low (see Appendix 7). |
| Were the same techniques and tools used for all participants? | 332 | 89% | |
| Number who enrolled in the study / number who were eligible for the study >=80% | 361 | 96% | |
| Total score for quality assessment | Total 19 | | Median 11, mean 11.4 |

## Appendix 10: Summary of included studies

Anti-HBc = hepatitis B core antibody, anti-HBs = hepatitis B surface antibody, HBsAg = hepatitis B surface antigen, HBV = hepatitis B virus, HCC = hepatocellular carcinoma, HCV = hepatitis C virus, HIV = human immunodeficiency virus.

| Author and year | Country | Population | Details if "other" | Study setting | Number of participants recruited to study | Number of participants negative for HBsAg | Number of participants tested for HBV DNA | Tissue tested | Serological criteria to test for OBI | Total OBI | OBI prevalence (total OBI / number tested for HBV DNA) |
|---|---|---|---|---|---|---|---|---|---|---|---|
| **Abbasi et al. 2016** | Iran | Blood Donors | | A blood transfusion centre in Ahvaz City, Iran | 184 | 184 | 184 | Blood | HBsAg - | 0 | 0.0% |
| **Abd El Kader Mahmoud et al. 2013** | Sudan | Blood Donors | | The central blood bank in Sudan | 100 | 100 | 42 | Blood | HBsAg -, anti-HBc + | 38 | 90.5% |
| **Aghakhani et al. 2010** | Iran | Haemodialysis patients | | Five dialysis units in Tehran, Iran | 289 | 281 | 18 | Blood | HBsAg -, anti-HBc + | 9 | 50.0% |
| **Aghasadeghi et al. 2014** | Iran | Others without liver disease/deranged LFTs | Non-responders to HBV vaccine | Ethical committee from Iranian Society for Support of Patients with Infectious Diseases | 52 | 52 | 52 | Blood | HBsAg - | 0 | 0.0% |
| **Aghayeva et al. 2018** | Azerbaijan | HCV positive patients | | | 194 | 170 | 170 | Blood | HBsAg -, anti-HBc + | 46 | 27.1% |
| **Aires et al. 2012** | Brazil | Others without liver disease/deranged LFTs | TB patients | Anuar Auad Hospital (the reference hospital for infectious diseases in Goiânia) | 402 | 389 | 90 | Blood | HBsAg -, anti-HBc + | 13 | 14.4% |
| **Akhoundi et al. 2015** | Iran | Healthcare workers | | 51st Annual Congress if the Iranian Dental Association | 1628 | 1621 | 52 | Blood | HBsAg -, anti-HBc + | 1 | 1.9% |
| **Al-Kubaisy et al. 2015** | Jordan | Blood Donors | | Blood Bank Unit Islamic Hospital | 578 | 578 | 30 | Blood | HBsAg -, anti-HBc + | 0 | 0.0% |
| **Al-Moslih et al. 2010** | United Arab Emirates/Saudi Arabia | Patients with liver impairment for any other reason | Chronic liver disease patients | United Arab Emirates | 248 | 159 | 159 | Blood | HBsAg - | 22 | 13.8% |
| **Alavian et al. 2011** | Iran | Haematology patients | Thalassemic and hemophilic patients with chronic HCV without HBsAg | Tehran hepatitis Centre (THC) | 145 | 145 | 145 | Blood | HBsAg - | 0 | 0.0% |
| **Alizadeh et al. 2014** | Iran | Blood Donors | | Blood transfusion centers in Tehran | 5000 | 5000 | 5000 | Blood | HBsAg - | 2 | 0.0% |

| | | | | | | | | | | | |
|---|---|---|---|---|---|---|---|---|---|---|---|
| **Alzahrani et al. 2019** | United Arab Emirates/Saudi Arabia | Blood Donors | | | 22842 | 22103 | 22103 | Blood | HBsAg - | 12 | 0.1% |
| **Ansari et al. 2015** | Iran | Cirrhotic patients, incl. cryptogenic/other aetiologies | Cryptogenic cirrhosis patients | the Imam Khomeini Hospital of Ahvaz | 38 | 27 | 27 | Blood | HBsAg - | 11 | 40.7% |
| **Anvari et al. 2014** | Iran | Cirrhotic patients, incl. cryptogenic/other aetiologies | Cryptogenic cirrhosis patients | Tehran Hepatitis Network | 29 | 29 | 29 | Blood | HBsAg - | 11 | 37.9% |
| **Araujo et al. 2011** | Brazil | Blood Donors | | COLSAN-Associação Beneficente de Coleta de Sangue (São Paulo, Brazil) | 250136 | 248761 | 4252 | Blood | HBsAg -, anti-HBc + | 36 | 0.8% |
| **Ashshi et al. 2012** | United Arab Emirates/Saudi Arabia | Blood Donors | | Hera'a General Hospital | 1000 | 1000 | 75 | Blood | HBsAg -, anti-HBc + | 2 | 2.7% |
| **Asim et al. 2010** | India | Blood Donors | | Blood bank of Lok Nayak Hospital, New Delhi | 2175 | 2175 | 413 | Blood | HBsAg -, anti-HBc + | 31 | 7.5% |
| **Asli et al. 2016** | Iraq | Others without liver disease/deranged LFTs | | Loghman Hospital in Tehran | 229 | | 59 | Blood | HBsAg -, anti-HBc + | 13 | 22.0% |
| **Athira et al. 2018** | India | Blood Donors | | Department of Transfusion Medicine and Microbiology, JIPMER, Puducherry | 1102 | 1102 | 156 | Blood | HBsAg -, anti-HBc + | 52 | 33.3% |
| **Attia et al. 2012** | Cote d'Ivoire | HIV positive patients | | Multiple centres in Abidjan, Cote D'Ivoire: (Integrated Center of Bioclinical Research of Abidjan, General Medicine Department of Yopougon Teaching Hospital, Department of Infectious and Tropical Diseases of Treichville Teaching Hospital, Integrated Center for Bio-Clinical Research in Treichville, Abidjan) | 491 | 425 | 188 | Blood | HBsAg -, anti-HBc + | 40 | 21.3% |
| **Ayatollahi et al. 2016** | Iran | Haemodialysis patients | | Hospitals of Yazd, Iran | 126 | 126 | 126 | Blood | HBsAg - | 1 | 0.8% |
| **Bae et al. 2012** | South Korea | Others without liver disease/deranged LFTs | Renal transplant patients | Samsung Medical Center | 377 | 291 | 217 | Blood | Other criteria | 5 | 2.3% |
| **Baghbanian et al. 2016** | Iran | Haematology patients | Patients with haematological and solid cancer | Yazd Shahid Sadoughi University School of Medicine | 204 | 204 | 204 | Blood | HBsAg -, anti-HBc + | 9 | 4.4% |
| **Bakaloudi et al. 2019** | Greece | Blood Donors | | AHEPA Center of molecular screening of blood donors (Greece) | 1755862 | 1753702 | 1753702 | Blood | HBsAg - | 228 | 0.0% |

| | | | | | | | | | | | |
|---|---|---|---|---|---|---|---|---|---|---|---|
| **Barth et al. 2010** | Netherlands | HIV positive patients | | University Medical Centre Utrecht | 846 | 733 | 259 | Blood | HBsAg -, anti-HBc + | 7 | 2.7% |
| **Barth et al. 2010** | South Africa | HIV positive patients | | Ndlovu Medical Centre | 248 | 241 | 62 | Blood | HBsAg -, anti-HBc + | 6 | 9.7% |
| **Belen Pisano et al. 2016** | Argentina | Blood Donors | | Fundacion Banco Central de Sangre in the city of Cordoba | 70102 | | 70067 | Blood | HBsAg - | 4 | 0.0% |
| **Bhattacharya et al. 2014** | India | Others without liver disease/deranged LFTs | Tribes - six aboriginal tribes on the Car Nicobar Islands of India | Two villages of Car Nicobar Islands | 612 | 558 | 558 | Blood | Other criteria | 62 | 11.1% |
| **Biswas et al. 2013** | India | Blood Donors | | Eastern India | 2195 | 2195 | 268 | Blood | HBsAg -, anti-HBc + | 65 | 24.3% |
| **Boavida et al. 2010** | Portugal | Blood Donors | | Lisbon Regional Blood Center | 221165 | 221077 | 221077 | Blood | HBsAg - | 3 | 0.0% |
| **Borzooy et al. 2015** | Iran | Healthcare workers | | 3 hospitals (Tehran Cardiac Center, Mirza Koochak Khan and Bahrami) | 120 | 120 | 120 | Blood | HBsAg - | 4 | 3.3% |
| **Cable et al. 2011** | South Africa | Blood Donors | | | 649754 | | 409 | Blood | HBsAg - | 13 | 3.2% |
| **Calvaruso et al. 2018** | Italy | HCV positive patients | HCV positive cirrhotic patients | Liver and GI Unit of University of Palermo | 104 | 96 | 96 | Blood | HBsAg - | 0 | 0.0% |
| **Capezzuto et al. 2010** | Italy | Blood Donors | | City of Parma and the surrounding province | 32608 | 32608 | 32608 | Blood | HBsAg - | 2 | 0.0% |
| **Cardoso et al. 2013** | Portugal | HCV positive patients | | | 100 | 100 | 100 | Liver | HBsAg - | 57 | 57.0% |
| **Cardoso et al. 2013** | Portugal | HCV positive patients | | | 100 | 100 | 100 | Blood | HBsAg - | 0 | 0.0% |
| **Carimo et al. 2018** | Mozambique | HIV positive patients | | Two public health centers from Maputo city, Mozambique | 518 | 471 | 206 | Blood | HBsAg -, anti-HBc + | 17 | 8.3% |
| **Cavalcanti de Albuquerque et al. 2012** | Brazil | Haemodialysis patients | | 5 clinics in Recife, Pernambuco | 752 | 752 | 201 | Blood | HBsAg -, anti-HBc + | 3 | 1.5% |
| **Cavaretto et al. 2018** | Brazil | Others without liver disease/deranged LFTs | Manicurists and their clients | Community in Campo Grande, MS, Central Brazil | 514 | 512 | 27 | Blood | HBsAg -, anti-HBc + | 1 | 3.7% |
| **Caviglia et al. 2012** | Italy | HCV positive patients | | | 35 | 35 | 35 | Liver | HBsAg - | 13 | 37.1% |
| **Caviglia et al. 2013** | Italy | Others without liver disease/deranged LFTs | Liver donors | Department of Medical Sciences, University of Torino, Turin, Italy | 56 | 56 | 56 | Liver | HBsAg - | 22 | 39.3% |
| **Caviglia et al. 2015** | Italy | Others without liver disease/deranged LFTs | | Medical Sciences and Surgical Sciences, University of Turin, Turin, Italy. Liver Transplant Center,Città della Salute e della Scienza, Molinette Hospital, Turin, Italy. Gastroenterology, Città della Salute e della Scienza, | 78 | 78 | 78 | Liver | HBsAg -, anti-HBc + | 44 | 56.4% |

| | | | | | | | | | | | |
|---|---|---|---|---|---|---|---|---|---|---|---|
| | | | | Molinette Hospital, Turin, Italy. | | | | | | | |
| **Caviglia et al. 2018** | Italy | Others without liver disease/deranged LFTs | | Liver Transplant Center of the University of Turin. | 100 | 100 | 100 | Blood | HBsAg -, anti-HBc + | 52 | 52.0% |
| **Chadwick et al. 2013** | United Kingdom | HIV positive patients | | HIV clinics in London (Royal Free Hospital), Newcastle (Royal Victoria Infirmary) and Middlesbrough (James Cook University Hospital) | 335 | 335 | 216 | Blood | HBsAg - | 14 | 6.5% |
| **Chandra et al. 2011** | India | HIV positive patients | | | 100 | 100 | 100 | Blood | HBsAg - | 6 | 6.0% |
| **Chang et al. 2013** | Taiwan | HCC patients | | Chang Gung Medical Center (CGMC) | 115 | 80 | 80 | Liver | HBsAg - | 16 | 20.0% |
| **Chen et al. 2012** | Taiwan | Haemodialysis patients | | | 158 | 133 | 133 | Blood | HBsAg - | 9 | 6.8% |
| **Chen et al. 2017** | Taiwan | HCV positive patients | | National Taiwan University Hospital | 183 | 183 | 183 | Blood | HBsAg -, anti-HBc + | 56 | 30.6% |
| **Chenari et al. 2014** | Iran | Others without liver disease/deranged LFTs | Asymptomatic healthy HTLV-I positive carriers | Department of Immunology, Ghaem Hospital, Mashhad | 109 | 109 | 109 | Blood | HBsAg - | 1 | 0.9% |
| **Cheung et al. 2011** | Hong Kong | Haematology patients | Lymphoma patients | United Christian Hospital, Hong Kong | 47 | 47 | 47 | Blood | Other criteria | 10 | 21.3% |
| **Chinchilla-Reyes et al. 2013** | USA | Others without liver disease/deranged LFTs | Organ/tissue donors | Mendez National Institute of Transplantation, Los Angeles | 4216 | 4179 | 4179 | Blood | HBsAg - | 5 | 0.1% |
| **Cho et al. 2016** | South Korea | Cirrhotic patients, incl. cryptogenic/other aetiologies | | Seoul National University Bundang Hospital | 47 | 47 | 47 | Blood | HBsAg - | 12 | 25.5% |
| **Cho et al. 2016** | South Korea | HCC patients | | Seoul National University Bundang Hospital | 44 | 44 | 44 | Blood | HBsAg - | 6 | 13.6% |
| **Costa et al. 2019** | Brazil | Others without liver disease/deranged LFTs | Leprosy patients | The outpatient dermatology clinic of a Reference Center for Infectious Diseases in Paraíba, in the Northeast region of Brazil | 403 | | 114 | Blood | Other criteria | 6 | 5.3% |
| **David et al. 2017** | India | HCC patients | | Departments of Gastroenterology and Hepatology, Christian Medical College Hospital, Vellore | 138 | 75 | 47 | Blood | HBsAg - | 0 | 0.0% |
| **de Castro Sant'Anna et al. 2018** | Brazil | Patients with liver impairment for any other reason | Unclear | Foundation Center of Hemotherapy and Hematology of Pará (HEMOPA) | 965 | | 181 | Blood | HBsAg -, anti-HBc +ve, anti-HBs - | 26 | 14.4% |

| | | | | | | | | | | | |
|---|---|---|---|---|---|---|---|---|---|---|---|
| **de Matos et al. 2013** | Brazil | Others without liver disease/deranged LFTs | | 34 public, private and charitable drug treatment centres in the capitals cities of three states (18 in Goiânia, state of Goiás, 8 in Campo Grande, state of Mato Grosso do Sul and 8 in Cuiabá, state of Mato Grosso) | 150 | 149 | 149 | Blood | HBsAg - | 19 | 12.8% |
| **Deng et al. 2012** | China | Blood Donors | | Dalian, China | 63393 | 63327 | 63327 | Blood | HBsAg - | 21 | 0.0% |
| **Dhawan et al. 2011** | India | Blood Donors | | | 1700 | 1700 | 100 | Blood | HBsAg -, anti-HBc + | 1 | 1.0% |
| **Diarra et al. 2018** | Burkina Faso | Others without liver disease/deranged LFTs | Unclear | Ouagadougou, at the Pietro Annigoni Biomolecular Research Center of Burkina Faso | 219 | 219 | 219 | Blood | HBsAg - | 56 | 25.6% |
| **Diniz et al. 2013** | Brazil | HCC patients | | Alfa Institute of Gastroenterology from Clinical Hospital | 49 | | 49 | Liver | HBsAg - | 1 | 2.0% |
| **Diniz et al. 2013** | Brazil | Patients with liver impairment for any other reason | Chronic liver disease patients without HCC | Alfa Institute of Gastroenterology from Clinical Hospital | 22 | | 22 | Liver | HBsAg - | 3 | 13.6% |
| **Dodd et al. 2018** | USA | Blood Donors | | American Red Cross blood donors | 22370271 | 22367284 | 22367284 | Blood | HBsAg - | 433 | 0.0% |
| **Dong et al. 2014** | China | Blood Donors | | Blood centre of Zhejiang province, China | 178447 | 78963 | 78963 | Blood | HBsAg - | 86 | 0.1% |
| **Dong et al. 2014** | China | Blood Donors | | Blood centre of Zhejiang province, China | 178447 | 99484 | 99484 | Blood | HBsAg - | 83 | 0.1% |
| **El Banna et al. 2017** | Lebanon | Blood Donors | | Transfusion center of a major hospital in Beirut, Lebanon | 7437 | 320 | 320 | Blood | HBsAg -, anti-HBc + | 1 | 0.3% |
| **El Makarem et al. 2012** | Egypt | Haemodialysis patients | | Dialysis unit of the Department of Internal Medicine, Minia University and the dialysis unit of the Department of Internal Medicine, Assuit University | 145 | 145 | 145 | Blood | HBsAg - | 6 | 4.1% |
| **El-Ghitany et al. 2013** | Egypt | Blood Donors | | Main blood banks in Alexandria | 256 | 254 | 254 | Blood | HBsAg - | 13 | 5.1% |
| **El-Ghitany et al. 2013** | Egypt | Others without liver disease/deranged LFTs | HCV +ve blood donors; not strictly people with "liver disease" but HCV +ve | Main blood banks in Alexandria | 256 | 254 | 254 | Blood | HBsAg - | 8 | 3.1% |
| **El-Maraghy et al. 2015** | Egypt | Patients with liver impairment for any other reason | Chronic liver disease patients | Internal medicine department, Faculty of Medicine, Suez Canal | 50 | 50 | 50 | Blood | HBsAg - | 3 | 6.0% |

| | | | | | | | | | | | |
|---|---|---|---|---|---|---|---|---|---|---|---|
| | | | | University (FOMSCU), Ismailia, Egypt | | | | | | | |
| **El-Sheredy et al. 2015** | Sudan | Haematology patients | Patients with haematological disorders | Medical Research Institute Hospital, Alexandria University. | 60 | 60 | 60 | Blood | HBsAg - | 32 | 53.3% |
| **Elbakush et al. 2014** | Libya | HIV positive patients | | The infectious disease department of Tripoli Medical Center and in the National Center Disease Control | 143 | 143 | 20 | Blood | HBsAg -, anti-HBc + | 3 | 15.0% |
| **Elbedewy et al. 2015** | Egypt | Haematology patients | Polytransfused patients | Hematology Unit, Internal Medicine Department, Faculty of Medicine, Tanta University | 79 | 79 | 12 | Blood | HBsAg -, anti-HBc +ve, anti-HBs - | 5 | 41.7% |
| **Elgohry et al. 2012** | Egypt | Haemodialysis patients | | Alexandria Main University Hospital Dialysis Unit | 93 | 93 | 93 | Blood | HBsAg - | 25 | 26.9% |
| **Elkady et al. 2017** | Egypt | Haematology patients | Patients with haematological malignancies | Oncology Department of the Sohag Faculty of Medicine and South Egypt Cancer Institution | 165 | 152 | 54 | Blood | HBsAg -, anti-HBc + | 23 | 42.6% |
| **Esmail et al. 2016** | Egypt | Haemodialysis patients | | Minia University Hospital (MUH) | 144 | 144 | 50 | Blood | HBsAg -, anti-HBc +ve, anti-HBs - | 12 | 24.0% |
| **Faro Stief et al. 2010** | Brazil | Others without liver disease/deranged LFTs | Prisoners | 3 prisons in Mato Grosso do Sul | 408 | 406 | 71 | Blood | HBsAg -, anti-HBc + | 0 | 0.0% |
| **Fernandez et al. 2010** | Argentina | Blood Donors | | | 260 | 111 | 111 | Blood | HBsAg -, anti-HBc + | 0 | 0.0% |
| **Ferrari et al. 2014** | Brazil | Others without liver disease/deranged LFTs | Liver donors (explanted livers) | Hospital das Clinicas, Universidade Federal de Minas Gerais, Belo Horizonte, MG, Brazil | 68 | 68 | 68 | Liver | HBsAg - | 3 | 4.4% |
| **Fontenele et al. 2015** | Brazil | Haemodialysis patients | | Clinical Research Center of the University Hospital at the Federal University of Maranhão (Hospital Universitário da Universidade Federal do Maranhão—HUUFMA), in the Central Public Health Laboratory of Maranhão-Oswaldo Cruz Institute (Laboratório Central de Saúde Pública do Maranhão-Instituto Oswaldo Cruz—LACEN), Brazil, and in | 301 | 301 | 301 | Blood | HBsAg - | 7 | 2.3% |

| | | | | | | | | | | | |
|---|---|---|---|---|---|---|---|---|---|---|---|
| | | | | two of the three dialysis units in São Luís, the capital of Maranhão state | | | | | | | |
| **Fopa et al. 2018** | Cameroon | Blood Donors | | Yaounde, Cameroon | 1167 | 1082 | 134 | Blood | HBsAg -, anti-HBc + | 4 | 3.0% |
| **Franz et al. 2013** | Brazil | Others without liver disease/deranged LFTs | Renal transplant patients | Renal Transplantation Unit of the Clementino Fraga Filho University Hospital, Rio de Janeiro, Brazil | 207 | 207 | 207 | Blood | HBsAg - | 2 | 1.0% |
| **Gachara et al. 2017** | Cameroon | HIV positive patients | | Mutengene Baptist Health Centre in the South West Region of Cameroon | 455 | 337 | 337 | Blood | HBsAg - | 20 | 5.9% |
| **Gad et al. 2011** | Egypt | Others without liver disease/deranged LFTs | Apparently healthy individuals prepared as potential liver donors | Gastroenterology Surgical Centre | 107 | 107 | 107 | Liver | HBsAg - | 21 | 19.6% |
| **Garcia-Montalvo et al. 2011** | Mexico | Blood Donors | | Central Blood Bank of the Ignacio Garcia Tellez National Medical Center, in Merida, Yucatan, Mexico | 20328 | | 372 | Blood | HBsAg -, anti-HBc + | 24 | 6.5% |
| **Giardina et al. 2013** | Italy | Others without liver disease/deranged LFTs | Rheumatology patients with inflammatory joint diseases | Academic Unit of Rheumatology at the University of Palermo | 57 | 53 | 53 | Blood | HBsAg - | 0 | 0.0% |
| **Gill et al. 2013** | Pakistan | Others without liver disease/deranged LFTs | Healthy patients applying for employment in gulf regions | | 100 | 100 | 100 | Blood | HBsAg - | 6 | 6.0% |
| **Gonzalez et al. 2010** | Spain | Blood Donors | | Spanish Red Cross Blood Transfusion Centre | 128212 | 128177 | 128177 | Blood | HBsAg - | 10 | 0.0% |
| **Gonzalez et al. 2010** | Spain | Blood Donors | | Transfusion Center of the Community of Madrid | 255,055 | 254897 | 254897 | Blood | HBsAg - | 5 | 0.0% |
| **Gouas et al. 2012** | Gambia | Cirrhotic patients, incl. cryptogenic/other aetiologies | Cirrhotic patients | Three tertiary hospitals sites in The Gambia | 78 | 34 | 34 | Blood | HBsAg - | 5 | 14.7% |
| **Gouas et al. 2012** | Gambia | HCC patients | | Three tertiary hospitals sites in The Gambia | 198 | 88 | 88 | Blood | HBsAg - | 21 | 23.9% |
| **Gouas et al. 2012** | Gambia | Others without liver disease/deranged LFTs | Hospital controls | Three tertiary hospitals sites in The Gambia | 325 | 274 | 274 | Blood | HBsAg - | 8 | 2.9% |
| **Grabarczyk et al. 2015** | Poland | Blood Donors | | Institute of Hematology and Transfusion Medicine (IHTM) | 421,447 | | 421447 | Blood | HBsAg - | 2 | 0.0% |
| **Grabarczyk et al. 2015** | Poland | Blood Donors | | Institute of Hematology and | 1,554,270 | | 1554270 | Blood | HBsAg - | 4 | 0.0% |

| | | | | | | | | | | | |
|---|---|---|---|---|---|---|---|---|---|---|---|
| | | | | Transfusion Medicine (IHTM) | | | | | | | |
| **Grabarczyk et al. 2015** | Poland | Blood Donors | | Institute of Hematology and Transfusion Medicine (IHTM) | 4,548,911 | | 4548911 | Blood | HBsAg - | 59 | 0.0% |
| **Grabarczyk et al. 2015** | Poland | Blood Donors | | Institute of Hematology and Transfusion Medicine (IHTM) | 3,426,077 | | 3426077 | Blood | HBsAg - | 59 | 0.0% |
| **Haghazali et al. 2011** | Iran | Haemodialysis patients | | Buali Hospital of Qazvin university of medical sciences | 134 | 129 | 129 | Blood | HBsAg - | 4 | 3.1% |
| **Hao et al. 2017** | China | HCV positive patients | | 86 of HCV infected patients from Guangzhou blood center and 193 of them from Yang-jiang prison drug addicts | 279 | 279 | 279 | Blood | HBsAg - | 12 | 4.3% |
| **Hashemi et al. 2015a** | Iran | Cirrhotic patients, incl. cryptogenic/other aetiologies | Cryptogenic cirrhosis patients | Gastroenterology and hepatology clinics of Ahvaz Jundishapur University | 50 | 50 | 50 | Blood | HBsAg - | 7 | 14.0% |
| **Hashemi et al. 2015a** | Iran | General population | | Gastroenterology and hepatology clinics of Ahvaz Jundishapur University | 80 | 80 | 80 | Blood | HBsAg - | 0 | 0.0% |
| **Hashemi et al. 2015b** | Iran | Haemodialysis patients | | | 82 | 82 | 82 | Blood | HBsAg - | 0 | 0.0% |
| **Hashemi et al. 2015b** | Iran | Haemodialysis patients | | Hemodialysis Unit at the Imam Khomeini Hospital | 82 | 82 | 82 | Blood | HBsAg - | 9 | 11.0% |
| **Hassan et al. 2011** | Egypt | HCC patients | | National Cancer Institute, Cairo University | 40 | 40 | 40 | Liver | HBsAg - | 25 | 62.5% |
| **Hassan et al. 2011** | Egypt | HCC patients | | National Cancer Institute, Cairo University | 40 | 40 | 40 | Blood | HBsAg - | 20 | 50.0% |
| **Hayashi et al. 2012** | Japan | Others without liver disease/deranged LFTs | IBD patients | | 107 | 107 | 107 | Blood | HBsAg - | 0 | 0.0% |
| **Helaly et al. 2015** | Egypt | Haemodialysis patients | | Hemodialysis Unit of theMedical Research Institute of Alexandria University | 100 | 100 | 100 | Blood | HBsAg - | 32 | 32.0% |
| **Hernandez et al. 2013** | USA | HCC patients | | Hawaii Tumor Registry | 61 | 44 | 44 | Liver | HBsAg - | 3 | 6.8% |
| **Hon-Lok Tang et al. 2016** | Hong Kong | Haemodialysis patients | | Dialysis unit of Princess Margaret Hospital, Hong Kong | 106 | 93 | 55 | Blood | HBsAg -, anti-HBc + | 1 | 1.8% |
| **Hong et al. 2015** | South Korea | Others without liver disease/deranged LFTs | Patients without liver disease | Sung-Shim local hospital (a center for primary care) in Seoul, South Korea | 87 | 87 | 87 | Blood | HBsAg - | 6 | 6.9% |

| | | | | | | | | | | | |
|---|---|---|---|---|---|---|---|---|---|---|---|
| **Huang et al. 2010** | China | Others without liver disease/deranged LFTs | Inpatients, not further specified | Changhai Hospital, Zhongshan Hospital, Changzheng Hospital, and Jinan Infectious Disease Hospital | 254 | 254 | 254 | Blood | HBsAg - | 15 | 5.9% |
| **Huang et al. 2010** | China | Patients with liver impairment for any other reason | Anti-HBc positive patients, unspecified | | 183 | 183 | 183 | Blood | HBsAg -, anti-HBc +ve, anti-HBs - | 3 | 1.6% |
| **Hudu et al. 2014** | Malaysia | Blood Donors | | | 1000 | 1000 | 55 | Blood | HBsAg -, anti-HBc + | 55 | 100.0% |
| **Huzmeli et al. 2018** | Turkey | Haemodialysis patients | | Cumhuriyet University HD, peritoneal dialysis and nephrology clinics | 200 | 200 | 200 | Blood | HBsAg - | 3 | 1.5% |
| **Iglecias et al. 2016** | Brazil | Others without liver disease/deranged LFTs | Prisoners | Prisons (Pres´ıdio de Transito de Campo Grande (PTCG), Instituto Penal de Campo Grande (IPCG) and Estabelecimento PenalJair Ferreira de Carvalho (EPJFC) Estabelecimento Penal Feminino Irma˜ Irma Zorzi (EPFIIZ)) | 216 | 213 | 19 | Blood | HBsAg -, anti-HBc + | 2 | 10.5% |
| **Jang et al. 2011** | South Korea | Patients with liver impairment for any other reason | Chronic liver disease patients | | 71 | 71 | 71 | Liver | HBsAg - | 23 | 32.4% |
| **Ji et al. 2011** | China | Blood Donors | | | 10080 | 9972 | 9972 | Blood | HBsAg -, anti-HBc + | 25 | 0.3% |
| **Jianguo et al. 2011** | China | HCC patients | | Tangdu Hospital and Xijing Hospital of the Fourth Military Medical University, and Xian Jiaotong University, Xian, China | 268 | 61 | 61 | Liver | HBsAg - | 17 | 27.9% |
| **Jianguo et al. 2011** | China | HCC patients | | Tangdu Hospital and Xijing Hospital of the Fourth Military Medical University, and Xian Jiaotong University, Xian, China | 268 | 61 | 61 | Blood | HBsAg - | 4 | 6.6% |
| **Jonaidi-Jafari et al. 2017** | Iran | Haematology patients | Patients with other coagulation factor deficiencies | Tehran hepatitis center | 15 | 15 | 15 | Blood | HBsAg - | 0 | 0.0% |
| **Jonaidi-Jafari et al. 2017** | Iran | Haematology patients | Patients with thalassemia | Tehran hepatitis center | 35 | 35 | 35 | Blood | HBsAg - | 0 | 0.0% |
| **Jonaidi-Jafari et al. 2017** | Iran | Haematology patients | Patients with haemophilia | Tehran hepatitis center | 95 | 95 | 95 | Blood | HBsAg - | 0 | 0.0% |
| **Kalantari et al. 2016** | Iran | Haemodialysis patients | | Nephrology and dialysis units in Kashani, Faiz and | 400 | 400 | 10 | Blood | HBsAg -, anti-HBc +ve, anti-HBs - | 0 | 0.0% |

| | | | | | | | | | | |
|---|---|---|---|---|---|---|---|---|---|---|
| | | | | Al-Zahra Hospitals in Isfahan, Iran | | | | | | |
| **Kang et al. 2010** | South Korea | Haemodialysis patients | | | 161 | 161 | 161 | Blood | HBsAg - | 4 | 2.5% |
| **Kang et al. 2010** | South Korea | Patients with liver impairment for any other reason | Anti-HBc positive patients, unspecified | Department of Laboratory Medicine, The East-West Neo Medical Center, School of Medicine, Kyung-Hee University, Seoul, Korea | 17677 | 16796 | 230 | Blood | HBsAg -, anti-HBc + | 4 | 1.7% |
| **Karaosmanoglu et al. 2013** | Turkey | HIV positive patients | | 4 centres following-up HIV patients in Istanbul | 567 | 321 | 29 | Blood | HBsAg -, anti-HBc +ve, anti-HBs - | 3 | 10.3% |
| **Karimi et al. 2016** | Iran | Blood Donors | | 2 main blood collection centres (Kermanshah and Ahwaz) | 86182 | 86080 | 2031 | Blood | HBsAg -, anti-HBc +ve, anti-HBs - | 0 | 0.0% |
| **Katayama et al. 2015** | Japan | Haemodialysis patients | | 9 dialysis centers in Hiroshima Prefecture, Japan | 1860 | 1812 | 1812 | Blood | HBsAg - | 2 | 0.1% |
| **Keechilot et al. 2016** | India | Blood Donors | | A blood bank in Kerala | 24338 | 24292 | 24214 | Blood | HBsAg - | 4 | 0.0% |
| **Khatoon et al. 2016** | Pakistan | HCV positive patients | | Fauji Foundation Hospital, Rawalpindi | 105 | 105 | 105 | Blood | HBsAg - | 8 | 7.6% |
| **Khil et al. 2013** | Ukraine | Haemodialysis patients | | Ukraine | 1080 | 914 | 46 | Blood | HBsAg -, anti-HBc +ve, anti-HBs - | 16 | 34.8% |
| **Kim et al. 2012** | South Korea | Blood Donors | | Hanmaeum Blood Center | 149471 | | 149310 | Blood | HBsAg - | 23 | 0.0% |
| **Kim et al. 2013** | South Korea | Others without liver disease/deranged LFTs | Outpatients, not further specified | Jeju National University Hospital and one other hospital in South Korea | 624 | 624 | 624 | Blood | HBsAg - | 41 | 6.6% |
| **Kim et al. 2015** | South Korea | Haemodialysis patients | | Artificial Kidney Unit of 2 referral hospitals, Jeju National University Hospital and Dankook University Hospital | 153 | 153 | 153 | Blood | HBsAg - | 2 | 1.3% |
| **Kim et al. 2015** | South Korea | Others without liver disease/deranged LFTs | Apparently healthy people receiving routine health checks per Korea National Health Policy | Artificial Kidney Unit of 2 referral hospitals, Jeju National University Hospital and Dankook University Hospital | 121 | 121 | 121 | Blood | HBsAg - | 3 | 2.5% |
| **Kim et al. 2017** | South Korea | Patients with liver impairment for any other reason | Unclear | | 1060 | 1060 | 1060 | Blood | HBsAg -, anti-HBc +ve, anti-HBs - | 42 | 4.0% |
| **Kishk et al. 2015** | Egypt | Blood Donors | | "Northeastern Egypt", not further specified | 126 | 44 | 44 | Blood | HBsAg -, anti-HBc + | 10 | 22.7% |

| | | | | | | | | | | | |
|---|---|---|---|---|---|---|---|---|---|---|---|
| **Kopacz et al. 2012** | Poland | Haematology patients | Patients at the Institute of Haematology and Transfusion | Institute of Hematology and Transfusion Medicine | 468 | 452 | 452 | Blood | HBsAg - | 28 | 6.2% |
| **Kopacz et al. 2017** | Poland | Blood Donors | | Blood centres in Poland | 12513283 | | 12502764 | Blood | HBsAg - | 151 | 0.0% |
| **Kucharska et al. 2016** | Poland | Haematology patients | HCV-infected haemophiliacs | Polish Coagulopathy Centres | 71 | 64 | 64 | Blood | HBsAg - | 0 | 0.0% |
| **Kwon et al. 2010** | South Korea | Cirrhotic patients, incl. cryptogenic/other aetiologies | Cirrhotic patients (non-alcoholic) | Gachon Unviersity Gil Hospital, Inchon, Korea | 17 | 17 | 17 | Blood | HBsAg - | 2 | 11.8% |
| **Kwon et al. 2010** | South Korea | HCC patients | | Gachon Unviersity Gil Hospital, Inchon, Korea | 10 | 10 | 10 | Blood | HBsAg - | 2 | 20.0% |
| **La et al. 2014** | South Korea | Patients with liver impairment for any other reason | Cirrhotic or HCC patients | | 94 | 94 | 94 | Blood | HBsAg - | 2 | 2.1% |
| **La et al. 2014** | South Korea | Patients with liver impairment for any other reason | Cirrhotic or HCC patients | | 114 | 114 | 114 | Blood | HBsAg - | 3 | 2.6% |
| **Lago et al. 2015** | Brazil | Others without liver disease/deranged LFTs | Afro-Brazilian community within Brazil | Furnas dos Dionisios (FD), a semi-isolated Afro-descendant community in Central Brazil | 139 | 131 | 26 | Blood | HBsAg -, anti-HBc + | 3 | 11.5% |
| **Lai et al. 2015** | Taiwan | General population | | Epidemiological study for vaccine preventable disease | 705 | 705 | 705 | Blood | HBsAg -, anti-HBc +ve, anti-HBs - | 38 | 5.4% |
| **Lai et al. 2016** | Taiwan | Others without liver disease/deranged LFTs | Completely HBV immunized individuals negative for anti-hepatitis B core antibody. | Chang-Gung Memorial Hospital | 30 | 30 | 30 | Blood | HBsAg - | 2 | 6.7% |
| **Lam et al. 2013** | Malaysia | Blood Donors | | National Blood Centre, Kuala Lumpur | 222082 | 221643 | 221643 | Blood | HBsAg - | 63 | 0.0% |
| **Lam et al. 2013** | Malaysia | Blood Donors | | National Blood Centre, Kuala Lumpur | 279885 | 279193 | 279193 | Blood | HBsAg - | 34 | 0.0% |
| **Langat et al. 2018** | Kenya | Blood Donors | | Six Regional Blood Transfusion Centres | 1006 | 1006 | 127 | Blood | HBsAg -, anti-HBc + | 0 | 0.0% |
| **Launay et al. 2011** | France | Patients with liver impairment for any other reason | Unclear | Cochin Hospital clinical microbiology laboratory | 6431 | 6112 | 349 | Blood | HBsAg -, anti-HBc +ve, anti-HBs - | 8 | 2.3% |
| **Le Naht Minh et al. 2016** | Vietnam | Blood Donors | | Cho Ray Blood Transfusion Centre | 27270 | 26620 | 26620 | Blood | HBsAg - | 32 | 0.1% |
| **Leetrakool et al. 2019** | Thailand | Blood Donors | | Chiang Mai University Hospital | 59380 | | 58703 | Blood | HBsAg - | 40 | 0.1% |
| **Leetrakool et al. 2019** | Thailand | Blood Donors | | Chiang Mai University Hospital | 55799 | | 54304 | Blood | HBsAg - | 153 | 0.3% |
| **Lelie et al. 2013** | Mixed | Blood Donors | | South-East Asia (Singapore, Hong Kong, Malaysia), Egypt, South Africa, | 10981776 | 10973148 | 10973148 | Blood | HBsAg - | 583 | 0.0% |

| | | | | | | | | | | | |
|---|---|---|---|---|---|---|---|---|---|---|---|
| | | | | Mediterranean (Italy, Spain), Central-North Europe (Switzerland, SLovenia, Poland, Finland, Denmark, Ireland), South Pacific (Australia, New Zealand). | | | | | | | |
| **Lelie et al. 2017** | Egypt | Blood Donors | | Egypt | 80631 | 79782 | 79782 | Blood | HBsAg - | 8 | 0.0% |
| **Lelie et al. 2017** | Mixed | Blood Donors | | Oceania (Australia and New Zealand) | 152961 | 152838 | 152838 | Blood | HBsAg - | 5 | 0.0% |
| **Lelie et al. 2017** | Mixed | Blood Donors | | Mediterranean (Italy and Spain) | 290042 | 289484 | 289484 | Blood | HBsAg - | 30 | 0.0% |
| **Lelie et al. 2017** | Mixed | Blood Donors | | North and Central Europe (Switzerland, Slovenia, Finland, Denmark, Ireland, Poland) | 294367 | 293923 | 293923 | Blood | HBsAg - | 5 | 0.0% |
| **Lelie et al. 2017** | Mixed | Blood Donors | | South East Asia (Singapore, Hong Kong, Malaysia) | 324665 | 321270 | 321270 | Blood | HBsAg - | 48 | 0.0% |
| **Lelie et al. 2017** | South Africa | Blood Donors | | South Africa | 360818 | 358085 | 358085 | Blood | HBsAg - | 117 | 0.0% |
| **Levi et al. 2013** | Brazil | Blood Donors | | Hospital Israelita Albert Einstein (HIAE) blood bank | 24441 | 24412 | 24412 | Blood | HBsAg - | 7 | 0.0% |
| **Li Cavoli et al. 2017** | Italy | Haemodialysis patients | | Palermo, Italy | 48 | 48 | 48 | Blood | HBsAg - | 0 | 0.0% |
| **Liang et al. 2010** | China | Others without liver disease/deranged LFTs | HIV patients that were mostly HCV +ve, too | Shanghai Public Health Clinical Center | 97 | 97 | 92 | Blood | HBsAg - | 27 | 29.3% |
| **Lin et al. 2016a** | China | Blood Donors | | Jiangsu Province Blood Center | 157119 | 154791 | 154791 | Blood | HBsAg - | 81 | 0.1% |
| **Liu et al. 2010** | China | Blood Donors | | Nanjing Red Cross Blood Center | 2972 | 2972 | 2972 | Blood | HBsAg - | 5 | 0.2% |
| **Liu et al. 2016b** | China | Blood Donors | | NAT program launched by Chinese National Health from 2010 | 20084187 | 20084187 | 20084187 | Blood | HBsAg - | 13551 | 0.1% |
| **Lok et al. 2011** | USA | HCC patients | | HALT-C trials | 91 | 91 | 28 | Liver | HBsAg - | 3 | 10.7% |
| **Lok et al. 2011** | USA | HCC patients | | HALT-C trials | 91 | 91 | 91 | Blood | HBsAg - | 0 | 0.0% |
| **Louisirirotchanakul et al. 2011** | Thailand | Blood Donors | | National Blood Centre, Thai Red Cross Society | 486,676 | 486676 | 486676 | Blood | HBsAg - | 71 | 0.0% |
| **Louisirirotchanakul et al. 2011** | Thailand | Blood Donors | | National Blood Centre, Thai Red Cross Society | 486,676 | 486676 | 486676 | Blood | HBsAg - | 104 | 0.0% |
| **Lovrantova et al. 2011** | Slovakia | Blood Donors | | 4 large centres from different regions of Slovakia | 65010 | 65010 | 61 | Blood | HBsAg -, anti-HBc + | 15 | 24.6% |
| **Magvansuren et al. 2015** | Mongolia | Blood Donors | | NCTM in Ulaanbaatar | 16700 | 14948 | 14948 | Blood | HBsAg - | 35 | 0.2% |
| **Maheswari et al. 2012** | India | Blood Donors | | Department of Transfusion Medicine, | 9100 | 8901 | 820 | Blood | HBsAg -, anti-HBc + | 2 | 0.2% |

| | | | | | | | | | | | |
|---|---|---|---|---|---|---|---|---|---|---|---|
| | | | | The Tamilnadu Dr. MGR Medical University, Guindy, Chennai, India | | | | | | | |
| **Mahmoud et al. 2016** | Egypt | HCV positive patients | | Hepatology outpatient department in Medical Research Institute, Alexandria, Egypt | 100 | 100 | 100 | Blood | HBsAg - | 18 | 18.0% |
| **Makroo et al. 2011** | India | Blood Donors | | Indraprastha Apollo Hospitals | 94247 | 93113 | 8660 | Blood | HBsAg -, anti-HBc +ve, anti-HBs - | 13 | 0.2% |
| **Makvandi et al. 2014** | Iran | Patients with liver impairment for any other reason | Unclear | Patients from different regions in Khuzestan province who attended Danesh Medical Laboratory in Ahvaz, Iran | 120 | 120 | 120 | Blood | HBsAg - | 12 | 10.0% |
| **Mardian et al. 2017** | Indonesia | Blood Donors | | Dr. Sardjito Hospital, Yogyakarta, Indonesia | 456 | 456 | 122 | Blood | Other criteria | 17 | 13.9% |
| **Margaritis et al. 2011** | Australia | Blood Donors | | Australian Red Cross Blood Service | 421272 | 421209 | 421209 | Blood | HBsAg - | 15 | 0.0% |
| **Marinho et al. 2014** | Brazil | Others without liver disease/deranged LFTs | Recyclable waste pickers<br>OBI was tested for HBsAg-neg anti-HBc positive participants | 15 recycling cooperatives in Goiânia City, Brazil | 431 | 428 | 52 | Blood | HBsAg -, anti-HBc + | 1 | 1.9% |
| **Mbangiwa et al. 2018** | Botswana | Others without liver disease/deranged LFTs | HIV +ve pregnant women | From the completed Tshipidi study | 384 | 372 | 299 | Blood | HBsAg - | 17 | 5.7% |
| **Mbangiwa et al. 2018** | Botswana | Pregnant women | HIV -ve pregnant women | From the completed Tshipidi study | 368 | 364 | 323 | Blood | HBsAg - | 24 | 7.4% |
| **Meilani et al. 2016** | Indonesia | Pregnant women | | Perak Public Health Centre, Surabaya, East Java, Indonesia | 193 | 50 | 50 | Blood | HBsAg -, anti-HBc + | 9 | 18.0% |
| **Meshi et al. 2014** | United Arab Emirates/Saudi Arabia | Blood Donors | | Central blood bank in Jazan province in Saudi Arabia | 8099 | 7983 | 7983 | Blood | HBsAg - | 9 | 0.1% |
| **Mhalla et al. 2017** | Tunisia | Haemodialysis patients | | Haemodialysis unit of the Nephrology Department (F. Bourguiba Teaching Hospital of Monastir) | 109 | 105 | 5 | Blood | HBsAg -, anti-HBc +ve, anti-HBs - | 2 | 40.0% |
| **Min et al. 2013** | South Korea | Others without liver disease/deranged LFTs | | Bucheon St. Mary’s Hospital (Participants from = the Incheon Probation & Parole Office Monitoring Rehabilitation Program (Incheon, Kyungki-do) | 318 | 297 | 297 | Blood | HBsAg - | 16 | 5.4% |
| **Mina et al. 2010** | Greece | Haemodialysis patients | | Six Dialysis Units in Central Greece | 366 | 346 | 346 | Blood | HBsAg - | 3 | 0.9% |

| | | | | | | | | | | | |
|---|---|---|---|---|---|---|---|---|---|---|---|
| **Minuk et al. 2014** | Canada | General population | | Epidemiological (serological) survey of HBV infection in the Canadian North | 1007 | 1007 | 1007 | Blood | HBsAg - | 55 | 5.5% |
| **Mitsumoto-Kaseida et al. 2019** | Japan | HIV positive patients | | Kyushu University Hospital | 147 | 126 | 119 | Blood | HBsAg - | 9 | 7.6% |
| **Mohanraj et al. 2015** | India | Patients with liver impairment for any other reason | Chronic liver disease patients | Kilpauk Medical College | 132 | 132 | 132 | Blood | HBsAg -, anti-HBc +ve, anti-HBs - | 8 | 6.1% |
| **Mok et al. 2017** | China | Others without liver disease/deranged LFTs | Rheumatology patients | | 310 | 299 | 105 | Blood | HBsAg -, anti-HBc + | 9 | 8.6% |
| **Monari et al. 2016** | Italy | General population | Routine screening as part of study evaluating the effect of introducing the HBV vaccine in Italy in 1991. No further information on setting given. | Routine screening as part of study evaluating the effect of introducing the HBV vaccine in Italy in 1991. No further information on setting given. | 2,454 | 86 | 9 | Blood | HBsAg -, anti-HBc + | 2 | 22.2% |
| **Moresco et al. 2014** | Brazil | Blood Donors | | Collection and Transfusion Units co-ordinated by FHEMOAM (The Hematology and Hemotherapy State of Amazonas Foundation), Manaus | 3600 | 3564 | 291 | Blood | HBsAg -, anti-HBc + | 8 | 2.7% |
| **Mori et al. 2011** | Japan | Others without liver disease/deranged LFTs | Rheumatoid arthritis patients | Clinical Research Center for Rheumatic Disease, Department of Rheumatology, NHO Kumamoto Saishunsou National Hospital | 239 | 237 | 237 | Blood | HBsAg - | 2 | 0.8% |
| **Mortensen et al. 2016** | USA | Others without liver disease/deranged LFTs | Veterans undergoing dental procedures | two Veterans Affairs medical center | 1891 | 1887 | 273 | Blood | HBsAg -, anti-HBc + | 6 | 2.2% |
| **Motta et al. 2010** | Brazil | Haemodialysis patients | | 1hemodialysis center in Rio de Janeiro, Brazil | 100 | 100 | 100 | Blood | HBsAg - | 15 | 15.0% |
| **Msomi et al. 2019** | South Africa | Haemodialysis patients | | Two hospital HD units in Durban, South Africa | 86 | 86 | 85 | Blood | HBsAg - | 9 | 10.6% |
| **Muche et al. 2018** | Germany | Others without liver disease/deranged LFTs | Haemodialysis and renal transplant patients | | 834 | 777 | 777 | PBMC | HBsAg - | 0 | 0.0% |
| **Muche et al. 2018** | Germany | Others without liver disease/deranged LFTs | Haemodialysis and renal transplant patients | | 834 | 777 | 777 | Blood | HBsAg - | 1 | 0.1% |

| | | | | | | | | | | | |
|---|---|---|---|---|---|---|---|---|---|---|---|
| **Muselmani et al. 2013** | Syria | Blood Donors | | Blood Transfusion Center of Damascus University | 1939 | 1913 | 31 | Blood | HBsAg -, anti-HBc +ve, anti-HBs - | 5 | 16.1% |
| **Muselmani et al. 2014** | Syria | Blood Donors | | Different blood transfusion centers in all Syrian geographic areas | 3896 | 3830 | 402 | Blood | HBsAg -, anti-HBc +ve, anti-HBs - | 5 | 1.2% |
| **Muto et al. 2018** | Japan | HCC patients | | Kyushu University Hospital | 90 | 75 | 60 | Liver | HBsAg - | 18 | 30.0% |
| **N'Dri-Yoman et al. 2010** | Cote d'Ivoire | HIV positive patients | | Four HIV Outpatient Clinics in Abdijan | 495 | 432 | 209 | Blood | HBsAg -, anti-HBc +ve, anti-HBs - | 51 | 24.4% |
| **Naderi et al. 2018** | Iran | Patients with liver impairment for any other reason | | Comprehensive Hemophilia Care Center in Iran | 100 | 100 | 100 | PBMC | HBsAg - | 3 | 3.0% |
| **Naderi et al. 2018** | Iran | Patients with liver impairment for any other reason | | Comprehensive Hemophilia Care Center in Iran | 100 | 100 | 100 | Blood | HBsAg - | 1 | 1.0% |
| **Nagakawa et al. 2013** | Japan | Haemodialysis patients | | Four haemodialysis units in Chiba, Japan | 1041 | 1035 | 212 | Blood | HBsAg -, anti-HBc + | 3 | 1.4% |
| **Ngonyolo et al. 2018** | South Africa | Others without liver disease/deranged LFTs | Patients, not further specified | Fuur provinces of South Africa (Gauteng, Limpopo, Northwest and Mpumalanga) | 80 | 78 | 39 | Blood | HBsAg -, anti-HBc +ve, anti-HBs - | 7 | 17.9% |
| **Ni et al. 2012** | Taiwan | General population | | Schools, institutes, or workplaces in Taipei City | 378 | | 71 | Blood | HBsAg -, anti-HBc + | 3 | 4.2% |
| **Niazi et al. 2015** | Pakistan | Blood Donors | | Armed Forces Institute of Transfusion (AFIT) in Rawalpindi, Northern Pakistan | 56772 | 56053 | 54438 | Blood | HBsAg - | 23 | 0.0% |
| **Nna et al. 2014** | Nigeria | Blood Donors | | Nnamdi Azikiwe University Teaching Hospital | 113 | 100 | 8 | Blood | HBsAg -, anti-HBc + | 8 | 100.0% |
| **Oliveira et al. 2016a** | Brazil | HIV positive patients | | Anuar Auad Hospital | 505 | 480 | 480 | Blood | HBsAg - | 19 | 4.0% |
| **Oliveira et al. 2016b** | Brazil | Others without liver disease/deranged LFTs | Men who have sex with men | Goiania, the capital of the State of Goias, Central Brazil | 522 | 517 | 517 | Blood | HBsAg - | 0 | 0.0% |
| **Olotu et al. 2016** | Nigeria | Blood Donors | | OAUTHC and SDAH blood banks | 507 | 502 | 354 | Blood | HBsAg -, anti-HBc + | 19 | 5.4% |
| **Oluyinka et al. 2015** | Nigeria | Blood Donors | | Two major blood banks in southwestern Nigeria | 429 | 429 | 429 | Blood | HBsAg - | 72 | 16.8% |
| **Omar et al. 2018** | Egypt | HCC patients | | Outpatient clinic at the Internal Medicine Department at the Suez Canal University Hospital, Ismailia, Egypt | 240 | 240 | 84 | Blood | HBsAg -, anti-HBc + | 20 | 23.8% |

| | | | | | | | | | | | |
|---|---|---|---|---|---|---|---|---|---|---|---|
| **Omar et al. 2018** | Egypt | HCV positive patients | | Outpatient clinic at the Internal Medicine Department at the Suez Canal University Hospital, Ismailia, Egypt | 240 | 240 | 52 | Blood | HBsAg -, anti-HBc + | 8 | 15.4% |
| **Ostankova et al. 2017** | Kazakhstan | Blood Donors | | | 500 | 500 | 500 | Blood | HBsAg - | 0 | 0.0% |
| **Ostankova et al. 2017** | Kazakhstan | Blood Donors | | | 500 | 500 | 500 | Blood | HBsAg - | 47 | 9.4% |
| **Ou et al. 2012** | China | Blood Donors | | Xiamen, China | 9188 | 9159 | 9159 | Blood | HBsAg - | 18 | 0.2% |
| **Ozdemir et al. 2017** | Turkey | Blood Donors | | Necmettin Erbakan University Meram Medical Faculty Blood Center | 3000 | 3000 | 3000 | Blood | HBsAg - | 9 | 0.3% |
| **Panigrahi et al. 2010** | India | Blood Donors | | The Red Cross blood bank in the city of Behrampur | 729 | 729 | 220 | Blood | HBsAg -, anti-HBc +ve, anti-HBs - | 66 | 30.0% |
| **Panigrahi et al. 2012** | India | HIV positive patients | | The Apex Referral Centre for HIV/AIDS at MedicalnCollege, Kolkata | 1077 | 112 | 38 | Blood | HBsAg -, anti-HBc + | 12 | 31.6% |
| **Papa et al. 2010** | Italy | Others without liver disease/deranged LFTs | IBD patients | Inpatients and outpatients affected by IBD seen in the Department of Internal Medicine and Gastroenterology, Catholic University of Rome, Italy | 301 | 300 | 22 | Blood | HBsAg -, anti-HBc + | 0 | 0.0% |
| **Pessoni et al. 2019** | Brazil | Haematology patients | Patients with haematological disorders | Two referral hospitals in Goiás, CentralBrazil | 322 | 318 | 41 | Blood | HBsAg -, anti-HBc + | 3 | 7.3% |
| **Pinato et al. 2012** | Italy | Haematology patients | Patients with haematological malignancies and healthy patients | | 240 | 229 | 229 | PBMC | HBsAg - | 15 | 6.6% |
| **Pollicino et al. 2019** | Italy | Patients with liver impairment for any other reason | Intrahepatic cholangiocarcinoma patients | Three Italian liver centres located in distinct geographic areas of the country (Milan, Rome, and Messina). | 47 | 47 | 47 | Liver | HBsAg - | 29 | 61.7% |
| **Punde et al. 2011** | India | Blood Donors | | | 1000 | 1000 | 1000 | Blood | HBsAg - | 22 | 2.2% |
| **Punde et al. 2011** | India | Healthcare workers | | | 100 | 100 | 100 | Blood | HBsAg - | 0 | 0.0% |
| **Punzalan et al. 2016** | Philippines | Blood Donors | | The Department of Health identified lead blood service facilities (BSF) | 3233 | 3221 | 3221 | Blood | HBsAg - | 48 | 1.5% |
| **Raimondo et al. 2018** | Italy | Patients with liver impairment for any other reason | Liver disease patients undergoing bariatric surgery | Two distinct liver centres (Trieste in Northern Italy, | 186 | 186 | 186 | Liver | HBsAg - | 24 | 12.9% |

| | | | | | | | | | | | |
|---|---|---|---|---|---|---|---|---|---|---|---|
| | | | | Messina in Southern Italy) | | | | | | | |
| **Ramezani et al. 2010a** | Iran | Blood Donors | | Blood Transfusion Organization in Tehran, Iran | 531 | 531 | 11 | Blood | HBsAg -, anti-HBc +ve, anti-HBs - | 0 | 0.0% |
| **Ramezani et al. 2010a** | Iran | Haemodialysis patients | | 5 dialysis units in Tehran | 289 | 289 | 18 | Blood | HBsAg -, anti-HBc +ve, anti-HBs - | 9 | 50.0% |
| **Ramezani et al. 2010a** | Iran | HIV positive patients | | Iranian Research Center for HIV/AIDS in Tehran, Iran | 106 | 106 | 22 | Blood | HBsAg -, anti-HBc +ve, anti-HBs - | 3 | 13.6% |
| **Ramezani et al. 2010b** | Iran | Others without liver disease/deranged LFTs | Mixture of haemodialysis and HIV +ve patients | | 395 | | 40 | Blood | HBsAg -, anti-HBc +ve, anti-HBs - | 12 | 30.0% |
| **Ramezani et al. 2015** | Iran | Haemodialysis patients | | a main di-alysis unit in Tehran, Iran unit in Tehran, Iran | 100 | 100 | 100 | Blood | HBsAg - | 1 | 1.0% |
| **Ranganathan et al. 2013** | India | Blood Donors | | Apollo Hospital Dialysis Unit | 21291 | 21291 | 21291 | Blood | HBsAg - | 10 | 0.0% |
| **Rastegarvand et al. 2015** | Iran | Haemodialysis patients | | Dialysis units of 4 hospitals in Ahvaz city from January 2012 to April 2012. | 203 | 203 | 203 | Blood | HBsAg -, anti-HBc + | 6 | 3.0% |
| **Ravula et al. 2018** | India | Others without liver disease/deranged LFTs | Deferred blood donors | | 1000 | 992 | 992 | Blood | HBsAg - | 1 | 0.1% |
| **Rinonce et al. 2013** | Indonesia | Haemodialysis patients | | A hemodialysis unit in Yogyakarta, Indonesia | 196 | 143 | 143 | Blood | HBsAg - | 21 | 14.7% |
| **Rinonce et al. 2013** | Indonesia | Healthcare workers | | Haemodialysis unit in Yogyakarta, Indonesia | 35 | 33 | 33 | Blood | HBsAg - | 0 | 0.0% |
| **Rizvi et al. 2014** | India | Patients with liver impairment for any other reason | Unclear | | 847 | 630 | 381 | Blood | HBsAg -, anti-HBc + | 29 | 7.6% |
| **Roman et al. 2010** | Mexico | Others without liver disease/deranged LFTs | Native communities | Nahuas community, Huichol community, and Mestizos community | 306 | 289 | 289 | Blood | HBsAg - | 41 | 14.2% |
| **Romano et al. 2013** | Italy | Blood Donors | | 21 Blood Transfusion Services in Italy (North, Central, South) | 46,147 | 31088 | 2186 | Blood | HBsAg -, anti-HBc + | 12 | 0.5% |
| **Rosa et al. 2017** | Brazil | HCV positive patients | | Clementino Fraga Filho University Hospital (HUCFF), Rio de Janeiro, Brazil | 151 | 151 | 151 | Blood | HBsAg - | 5 | 3.3% |
| **Sahu et al. 2018** | India | Patients with liver impairment for any other reason | Chronic liver disease patients | SCB Medical College, Cuttack and MKCG Medical College, Berhampur. | 554 | 353 | 79 | Blood | HBsAg -, anti-HBc +ve, anti-HBs - | 45 | 57.0% |
| **Said et al. 2013** | Egypt | Blood Donors | | 2 main blood transfusion centers | 3167 | 3167 | 303 | Blood | HBsAg -, anti-HBc + | 52 | 17.2% |

| | | | | | | | | | | | |
|---|---|---|---|---|---|---|---|---|---|---|---|
| **Saijo et al. 2015** | Japan | Haemodialysis patients | | | 161 | 157 | 41 | Blood | Other criteria | 2 | 4.9% |
| **Saitta et al. 2015** | Italy | HCC patients | | . All these cases had been included in previously published studies by the authors and were selected on the basis of the availability of amounts of stored tumor tissues sufficient to perform this further investigation. | 61 | 54 | 54 | Liver | HBsAg - | 34 | 63.0% |
| **Samiei et al. 2018** | Iran | Haemodialysis patients | | Golestan hospital in Ahvaz | 84 | 82 | 14 | Blood | HBsAg -, anti-HBc + | 4 | 28.6% |
| **Sang et al. 2011** | China | Blood Donors | | Shaoxing | 8692 | 8644 | 8644 | Blood | HBsAg - | 8 | 0.1% |
| **Saravanan et al. 2013** | India | General population | | National Referral Centre for Viral Hepatitis, Department of Microbiology, Faculty of Medicine, Dr ALM Post Graduate Institute of Basic Medical Sciences, University of Madras, and Chennai, India | 260 | 70 | 70 | Blood | HBsAg - | 3 | 4.3% |
| **Saravanan et al. 2013** | India | HCV positive patients | | National Referral Centre for Viral Hepatitis, Department of Microbiology, Faculty of Medicine, Dr ALM Post Graduate Institute of Basic Medical Sciences, University of Madras, and Chennai, India | 260 | 260 | 260 | Blood | HBsAg - | 19 | 7.3% |
| **Sauleda et al. 2011** | Spain | Blood Donors | | Blood bank in Catalonia | 123,527 | | 123282 | Blood | HBsAg - | 9 | 0.0% |
| **Sauleda et al. 2011** | Spain | Blood Donors | | Blood bank in Catalonia | 517,054 | | 517048 | Blood | HBsAg - | 49 | 0.0% |
| **Sav et al. 2010** | Turkey | Haemodialysis patients | | Erciyes University School of Medicine | 71 | 71 | 71 | Blood | HBsAg - | 12 | 16.9% |
| **Sav et al. 2010** | Turkey | Others without liver disease/deranged LFTs | Peritoneal dialysis patients | Erciyes University School of Medicine | 71 | 71 | 71 | Blood | HBsAg - | 7 | 9.9% |
| **Seferi et al. 2017** | Albania | Blood Donors | | | 20,810 | 20810 | 20810 | Blood | HBsAg - | 76 | 0.4% |
| **Selim et al. 2011** | Egypt | HCV positive patients | | Outpatient Clinics of National Liver Institute, Menoufiya, Egypt | 60 | 60 | 60 | Blood | HBsAg - | 23 | 38.3% |

| | | | | | | | | | | | |
|---|---|---|---|---|---|---|---|---|---|---|---|
| **Seo et al. 2011** | South Korea | Blood Donors | | Korean Red Cross Blood Center | 12461 | 1676 | 1676 | Blood | HBsAg -, anti-HBc + | 2 | 0.1% |
| **Shafiulla Sinha et al. 2016** | India | Haematology patients | Non-Hodgkin lymphoma patients | | 156 | 156 | 156 | Blood | HBsAg - | 23 | 14.7% |
| **Sharifi et al. 2013** | Iran | Blood Donors | | Sistan-o-Baluchistan | 3000 | 3000 | 300 | Blood | HBsAg -, anti-HBc + | 0 | 0.0% |
| **Sharifi et al. 2017** | Iran | HCV positive patients | | | 120 | 120 | 120 | Blood | HBsAg - | 0 | 0.0% |
| **Sheneef et al. 2012** | Egypt | HCV positive patients | | Departments of Medical Microbiology & Immunology, Clinical Pathology and Internal Medicine of Soahg University hospital, Egypt; patients were recruited from outpatient clinics of Internal Medicine Department, Sohag university hospital. | 60 | 60 | 60 | Blood | HBsAg - | 8 | 13.3% |
| **Shetty et al. 2011** | USA | Cirrhotic patients, incl. cryptogenic/other aetiologies | HCV +ve cirrhosis; explanted liver of liver transplant recipients | Inpatient and outpatient liver transplantation services at the Hospital of the University of Pennsylvania, Philadelphia, PA, USA. | 56 | 44 | 44 | Liver | HBsAg - | 22 | 50.0% |
| **Shetty et al. 2011** | USA | Patients with liver impairment for any other reason | HCV +ve cirrhosis liver transplant recipients | Inpatient and outpatient liver transplantation services at the Hospital of the University of Pennsylvania, Philadelphia, PA, USA. | 56 | 56 | 56 | Blood | HBsAg - | 16 | 28.6% |
| **Shim et al. 2011** | South Korea | Healthcare workers | | Kyung Hee Medical Center | 1812 | 1755 | 389 | Blood | Other criteria | 0 | 0.0% |
| **Shim et al. 2017** | South Korea | Patients with liver impairment for any other reason | Chronic liver disease patients | National Cancer Center (NCC) Hospital, Korea | 162 | 162 | 130 | Liver | HBsAg -, anti-HBc +ve, anti-HBs - | 55 | 42.3% |
| **Simonova et al. 2013** | Bulgaria | HCV positive patients | | Military Medical Academy, Sofia, Bulgaria | 152 | 152 | 152 | Blood | HBsAg - | 23 | 15.1% |
| **Simonova et al. 2016** | Bulgaria | Cirrhotic patients, incl. cryptogenic/other aetiologies | | Gastroenterology Clinic, Military Medical Academy – Sofia | 35 | 35 | 35 | Liver | HBsAg - | 16 | 45.7% |

| | | | | | | | | | | | |
|---|---|---|---|---|---|---|---|---|---|---|---|
| **Simonova et al. 2016** | Bulgaria | HCC patients | | Gastroenterology Clinic, Military Medical Academy – Sofia | 19 | 19 | 19 | Liver | HBsAg - | 11 | 57.9% |
| **Simonova et al. 2016** | Bulgaria | HCV positive patients | | Gastroenterology Clinic, Military Medical Academy – Sofia | 42 | 42 | 42 | Liver | HBsAg - | 12 | 28.6% |
| **Singh et al. 2016** | India | General population | | Coastal Odisha | 173 | 173 | 173 | Blood | HBsAg - | 9 | 5.2% |
| **Soedarmono et al. 2010** | Indonesia | Blood Donors | | | 7913 | 7913 | 507 | Blood | HBsAg -, anti-HBc + | 105 | 20.7% |
| **Sofian et al. 2012** | Iran | Others without liver disease/deranged LFTs | | Arak City, Iran | 153 | 142 | 11 | Blood | HBsAg -, anti-HBc + | 0 | 0.0% |
| **Sondlane et al. 2016** | South Africa | Healthcare workers | | Gauteng and Mpumalanga provinces. | 314 | 305 | 305 | Blood | HBsAg - | 21 | 6.9% |
| **Sood et al. 2016** | India | Others without liver disease/deranged LFTs | Blood donors with alcohol dependence | Tertiary centre, India | 369 | 369 | 369 | Blood | HBsAg - | 12 | 3.3% |
| **Sosa-Jurado et al. 2016** | Mexico | Blood Donors | | 10 sampling sites distributed throughout the state of Puebla, Mexico and blood bank of the national health centre "Manuel Avila Camacho" (Instituto Mexicano del Seguro Social) | 120,552 | | 156 | Blood | HBsAg -, anti-HBc + | 27 | 17.3% |
| **Sosa-Jurado et al. 2018** | Mexico | Others without liver disease/deranged LFTs | Gastroenterology patients | Gastroenterology Service of National Health Centre, Mexico | 116 | 116 | 45 | Blood | HBsAg -, anti-HBc + | 29 | 64.4% |
| **Sosa-Jurado et al. 2018** | Mexico | Others without liver disease/deranged LFTs | Gastroenterology patients | Gastroenterology Service of National Health Centre, Mexico | 116 | 116 | 45 | Blood | HBsAg -, anti-HBc + | 34 | 75.6% |
| **Souza et al. 2012** | Brazil | Pregnant women | | Four public maternity hospitals of São Luís | 541 | | 35 | Blood | HBsAg -, anti-HBc + | 0 | 0.0% |
| **Sowole et al. 2015** | United Kingdom | Haemodialysis patients | | The Royal Free Hospital and its satellite dialysis units | 793 | 778 | 138 | Blood | HBsAg -, anti-HBc + | 3 | 2.2% |
| **Spreafico et al. 2012** | Italy | Blood Donors | | | 28 | | 28 | Blood | HBsAg -, anti-HBc + | 12 | 42.9% |
| **Spreafico et al. 2012** | Italy | Blood Donors | | | 28 | | 28 | Blood | HBsAg -, anti-HBc + | 16 | 57.1% |
| **Spreafico et al. 2015** | Italy | Blood Donors | | Alessandro Manzoni Hospital's Department of Transfusion Medicine in Lecco, Italy | 97214 | 97213 | 97213 | Blood | HBsAg - | 18 | 0.0% |
| **Squadrito et al. 2013** | Italy | HCV positive patients | | Division of Clinical and Molecular Hepatology, University of Messina, Messina, Italy | 326 | 326 | 326 | Liver | HBsAg - | 128 | 39.3% |
| **Stolz et al. 2010** | Switzerland | Blood Donors | | Blood Transfusion Service SRC Berne | 306,000 | 305976 | 305976 | Blood | HBsAg - | 6 | 0.0% |
| **Su et al. 2011** | Taiwan | Blood Donors | | Taipei Blood Centre | 10824 | 10727 | 10727 | Blood | HBsAg - | 12 | 0.1% |

| | | | | | | | | | | | |
|---|---|---|---|---|---|---|---|---|---|---|---|
| **Suo et al. 2019** | China | Blood Donors | | Quinghai Blood Centre | 750 | 750 | 750 | Blood | HBsAg - | 8 | 1.1% |
| **Sykes et al. 2018** | South Africa | Blood Donors | | | 91540 | 91357 | 91357 | Blood | HBsAg - | 18 | 0.0% |
| **Taha et al. 2013** | Egypt | HCC patients | | Internal Medicine Clinic of Ain Shams University (Cairo, Egypt) | 40 | 20 | 20 | Liver | HBsAg - | 18 | 90.0% |
| **Taha et al. 2013** | Egypt | HCC patients | HCV + HCC | Internal Medicine Clinic of Ain Shams University (Cairo, Egypt) | 40 | 20 | 20 | Blood | HBsAg - | 7 | 35.0% |
| **Taha et al. 2013** | Egypt | HCV positive patients | | Internal Medicine Clinic of Ain Shams University (Cairo, Egypt) | 40 | 20 | 20 | Liver | HBsAg - | 5 | 25.0% |
| **Taha et al. 2013** | Egypt | HCV positive patients | | Internal Medicine Clinic of Ain Shams University (Cairo, Egypt) | 40 | 20 | 20 | Blood | HBsAg - | 2 | 10.0% |
| **Taha et al. 2017** | Egypt | HCV positive patients | | Gastroenterology and hepatology department in the Suez Canal University Hospital, Ismailia, Egypt | 200 | 200 | 200 | Blood | HBsAg - | 17 | 8.5% |
| **Taira et al. 2013** | Japan | Blood Donors | | Tokyo Blood Center | 640,628 | 640628 | 4742 | Blood | Other criteria | 92 | 1.9% |
| **Taira et al. 2013** | Japan | Blood Donors | | Japanese Red Cross (JRC) blood centers | 8,746,037 | 8746037 | 8746037 | Blood | HBsAg - | 133 | 0.0% |
| **Taira et al. 2013** | Japan | Blood Donors | | Japanese Red Cross (JRC) blood centers | 19,513,054 | 19513054 | 19513054 | Blood | HBsAg - | 76 | 0.0% |
| **Taira et al. 2013** | Japan | Blood Donors | | Japanese Red Cross (JRC) blood centers | 24,702,784 | 24702784 | 24702784 | Blood | HBsAg - | 473 | 0.0% |
| **Takeda et al. 2013** | Japan | Patients with liver impairment for any other reason | Chronic hepatitis (HBsAg -ve) patients | The Researche Center for Hepatitis & Immunology, National Center for Global Health and Medicine, chiba, Japan | 84 | 84 | 84 | Blood | HBsAg - | 7 | 8.3% |
| **Tandoi et al. 2014** | Italy | Others without liver disease/deranged LFTs | | Liver Transplant Center of the University of Turin. | 70 | 70 | 70 | Liver | HBsAg - | 30 | 42.9% |
| **Tandoi et al. 2014** | Italy | Others without liver disease/deranged LFTs | | Liver Transplant Center of the University of Turin. | 70 | 70 | 70 | Blood | HBsAg - | 5 | 7.1% |
| **Tang et al. 2018** | China | Blood Donors | | Guangzhou blood transfusion centre | 1,732 | 1732 | 1732 | Blood | HBsAg -, anti-HBc + | 9 | 0.5% |
| **Tang et al. 2018** | China | Blood Donors | | Guangzhou blood transfusion centre | 14,937 | 14928 | 14928 | Blood | HBsAg - | 10 | 0.1% |
| **Thabit et al. 2012** | Yemen | Patients with liver impairment for any other reason | Chronic liver disease patients | | 280 | 236 | 22 | Blood | HBsAg -, anti-HBc +ve, anti-HBs - | 11 | 50.0% |

| | | | | | | | | | | | |
|---|---|---|---|---|---|---|---|---|---|---|---|
| **Thabit et al. 2017** | Egypt | HCV positive patients | | | 200 | 200 | 200 | Blood | HBsAg - | 21 | 10.5% |
| **Tirri et al. 2013** | Italy | Others without liver disease/deranged LFTs | Rheumatology patients | | 303 | | 50 | Blood | HBsAg -, anti-HBc + | 0 | 0.0% |
| **Tjempakasari et al. 2014** | Indonesia | Haemodialysis patients | | 4 hospitals in Surabaya | 136 | | 51 | Blood | HBsAg -, anti-HBc +ve, anti-HBs - | 4 | 7.8% |
| **Torres et al. 2012** | USA | Patients with liver impairment for any other reason | Patients with haematological disorders and abnormal liver function | | 18 | 18 | 18 | Blood | HBsAg - | 2 | 11.1% |
| **Tramuto et al. 2013a** | Italy | Others without liver disease/deranged LFTs | Immigrants of non-Italian ethnicity | Migration Medicine ambulatory of the University Hospital ‘‘P. Giaccone’’—Palermo (Italy) | 339 | 339 | 339 | Blood | HBsAg - | 11 | 3.2% |
| **Tramuto et al. 2013b** | Italy | HIV positive patients | | Infectious Diseases ward of the University Hospital P. Giaccone, Palermo, Italy | 405 | 145 | 145 | Blood | HBsAg - | 7 | 4.8% |
| **Trinks et al. 2010** | Argentina | Others without liver disease/deranged LFTs | | Buenos Aires | 80 | 64 | 64 | Blood | HBsAg -, anti-HBc + | 9 | 14.1% |
| **Trinks et al. 2010** | Argentina | Others without liver disease/deranged LFTs | | Buenos Aires | 80 | 66 | 66 | Blood | HBsAg -, anti-HBc + | 1 | 1.5% |
| **Tsaneva-Damyanova et al. 2018** | Bulgaria | Patients with liver impairment for any other reason | Liver disease patients without HCC | University Hospital St. Marina, Varna, Bulgaria | 78 | 78 | 78 | Blood | HBsAg -, anti-HBc + | 16 | 20.5% |
| **Tsoi et al. 2013** | Hong Kong | Blood Donors | | Hong Kong Red Cross Blood Transfusion Service | 399,326 | 398349 | 398349 | Blood | HBsAg - | 169 | 0.0% |
| **Tsoi et al. 2013** | Hong Kong | Blood Donors | | Hong Kong Red Cross Blood Transfusion Service | 517,072 | 515258 | 515258 | Blood | HBsAg - | 113 | 0.0% |
| **Us et al. 2017** | Turkey | Patients with liver impairment for any other reason | Hepatitis B patients | Eskisehir Osmangazi University Medical Faculty, Medical Microbiology Laboratory | 4036 | | 4036 | Blood | HBsAg - | 105 | 2.6% |
| **Vaezjalali et al. 2012** | Iran | Blood Donors | | | 300 | 300 | 25 | Blood | HBsAg -, anti-HBc + | 0 | 0.0% |
| **van de Laar et al. 2015** | Netherlands | Blood Donors | | | 311,259 | 311259 | 311259 | Blood | HBsAg - | 0 | 0.0% |
| **van de Laar et al. 2015** | Netherlands | Blood Donors | | | 70,914 | | 70883 | Blood | HBsAg - | 2 | 0.0% |
| **Varaklioti et al. 2017** | Greece | Haematology patients | Patients with inherited bleeding disorders (haemophilias A/B, von Willebrand disease, factor VII deficiency) | National Reference Center for Congenital Bleeding Disorders Disorders in Laiko General Hospital of Athens | 114 | 112 | 112 | Blood | HBsAg - | 2 | 1.8% |

| | | | | | | | | | | |
|---|---|---|---|---|---|---|---|---|---|---|
| **Velasquez et al. 2011** | Philippines | Haemodialysis patients | | Philippines-Philippine General Hospital | 120 | | 12 | Blood | HBsAg -, anti-HBc +ve, anti-HBs - | 2 | 16.7% |
| **Voiculescu et al. 2010** | Romania | Healthcare workers | | Romania | 93 | 91 | 91 | Blood | HBsAg - | 0 | 0.0% |
| **Voiculescu et al. 2010** | Romania | Others without liver disease/deranged LFTs | Contacts of hepatitis patients | Romania | 44 | 43 | 43 | Blood | HBsAg - | 0 | 0.0% |
| **Wang et al. 2015a** | China | Blood Donors | | 12 blood centers (Shanghai Blood Center [SH], Baoji Central Blood Bank [BJ], Chengdu Blood Center [CD], Changzhou Central Blood Bank [CZ], Jiangsu Province Blood Center [JS], Jiangxi Province Blood Center [JX], Qingdao Blood Center [QD], Blood Center of Shandong Province [SD], Tianshui Central Blood Bank [TS], Wuhan Blood Center [WH], Xianyang Central Blood Bank [XY], and Yancheng Center Blood Bank [YC]) | 826044 | 826044 | 667 | Blood | HBsAg - | 413 | 61.9% |
| **Wang et al. 2015b** | China | Blood Donors | | Blood donors in three Chinese cities. | 482370 | 368940 | 368940 | Blood | HBsAg -, anti-HBc +ve, anti-HBs - | 156 | 0.0% |
| **Wang et al. 2015c** | China | Haematology patients | Multiple myeloma patients | 5 grade-III hospitals (Xi'an Central Hospital, Shaanxi People's Hospita', Xi'an Jiaotong University Xibei Hospital, "so on") | 185 | 170 | 170 | Blood | HBsAg - | 6 | 3.5% |
| **Wang et al. 2015c** | China | Others without liver disease/deranged LFTs | Tumor-free patients | 5 grade-III hospitals (Xi'an Central Hospital, Shaanxi People's Hospita', Xi'an Jiaotong University Xibei Hospital, "so on") | 182 | 174 | 170 | Blood | HBsAg - | 1 | 0.6% |
| **Wang et al. 2016** | China | Blood Donors | | Shenzhen, Southern China | 569145 | 555949 | 475538 | Blood | HBsAg - | 106 | 0.0% |
| **Wang et al. 2017a** | China | HCV positive patients | | Humanity and Health Medical Centre, Hong | 327 | 317 | 317 | Blood | HBsAg - | 124 | 39.1% |

| | | | | | | | | | | | |
|---|---|---|---|---|---|---|---|---|---|---|---|
| | | | | Kong Special Administrative Region, China | | | | | | | |
| **Wang et al. 2017b** | China | Blood Donors | | Clinical Laboratory Jiangxi Blood center, Nanchang, China | 158232 | 158205 | 158205 | Blood | HBsAg - | 79 | 0.0% |
| **Watanabe et al. 2013** | Japan | Others without liver disease/deranged LFTs | SLE patients who were receiving or planned to receive immunosuppressive therapy | Department of Hematology and Rheumatology in Tohoku University hospital. | 248 | 246 | 41 | Blood | Other criteria | 1 | 2.4% |
| **Wolff et al. 2011** | Brazil | Blood Donors | | Eight blood centers in Porto Alegre, Southern Brazil: Hospital de Clínicas de Porto Alegre, Santa Casa de Misericórdia de Porto Alegre, Hospital Moinhos de Vento, Hospital Ernesto Dorneles, Laboratório Marques-Pereira, Hospital Nossa Sen-hora da Conceição, Hospital São Lucas, Hemocentro do Rio Grande do Sul | 534 | 158 | 158 | Blood | HBsAg - | 0 | 0.0% |
| **Wong et al. 2011** | Hong Kong | HCC patients | | Asian HCC patients in HK whom underwent liver resection. | 61 | 40 | 48 | Liver | HBsAg - | 30 | 62.5% |
| **Wong et al. 2019** | Hong Kong | HCC patients | | Queen Mary Hospital, Hong Kong | 90 | 90 | 90 | Liver | HBsAg - | 62 | 68.9% |
| **Wong et al. 2019** | Hong Kong | Patients with liver impairment for any other reason | | Queen Mary Hospital, Hong Kong | 20 | 20 | 20 | Liver | HBsAg - | 3 | 15.0% |
| **Wu et al. 2011** | China | Others without liver disease/deranged LFTs | Inpatients and outpatients, not further specified | Ruijin Hospital | 468 | 468 | 468 | Blood | HBsAg - | 69 | 14.7% |
| **XiaoKun et al. 2013** | China | Blood Donors | | Blood donors in Zhangjiakou | 5498 | 5417 | 5417 | Blood | HBsAg - | 13 | 0.2% |
| **Xie et al. 2012** | China | Blood Donors | | | 63351 | 84990 | 84990 | Blood | HBsAg - | 41 | 0.0% |
| **Xie et al. 2015** | China | Patients with liver impairment for any other reason | Patients undergoing LT for alcoholic hepatitis | Tianjin First Center Hospital | 43 | 43 | 43 | Liver | HBsAg - | 18 | 41.9% |
| **Xin et al. 2011** | China | Blood Donors | | Shenzhen Blood Centre | 165371 | 165371 | 165371 | Blood | HBsAg - | 22 | 0.0% |
| **Xu et al. 2010** | China | General population | | | 2919 | 2859 | 106 | Blood | Other criteria | 81 | 76.4% |
| **Xu et al. 2017a** | China | Blood Donors | | Baoji Blood Center | 110,843 | | 110843 | Blood | HBsAg - | 43 | 0.0% |
| **Xu et al. 2017b** | China | Patients with liver impairment for any other reason | Unclear | Research Center for Clinical and Translational | 130 | 120 | 100 | Liver | HBsAg -, anti-HBc + | 22 | 22.0% |

| | | | | | | | | | | | |
|---|---|---|---|---|---|---|---|---|---|---|---|
| | | | | Medicine, Beijing 302 Hospital | | | | | | | |
| **Xu et al. 2017c** | China | Blood Donors | | Five blood services in the Zhejiang Province, China | 230000 | 230000 | 230000 | Blood | HBsAg - | 234 | 0.1% |
| **Xu et al. 2017d** | China | Blood Donors | | | 157985 | | 157985 | Blood | HBsAg -, anti-HBc + | 39 | 0.0% |
| **Yamaji et al. 2018** | Japan | HCC patients | | Saga Prefecture | 257 | 257 | 257 | Liver | HBsAg - | 15 | 5.8% |
| **Yang et al. 2010** | Taiwan | Blood Donors | | Taipei Blood Center | 4210 | 4179 | 4179 | Blood | HBsAg - | 6 | 0.1% |
| **Yang et al. 2010** | Taiwan | Blood Donors | | Taipei Blood Center | 6080 | 6044 | 6044 | Blood | HBsAg - | 3 | 0.0% |
| **Yang et al. 2012** | China | Haematology patients | HBV positive non-Hodgkin lymphoma patients | West China Hospital | 846 | 418 | 25 | Blood | HBsAg -, anti-HBc + | 0 | 0.0% |
| **Yang et al. 2013** | China | Blood Donors | | Qingdao Blood Center | 10,967 | 10967 | 10967 | Blood | HBsAg - | 59 | 0.5% |
| **Yang et al. 2015** | Taiwan | HCC patients | | | 170 | 170 | 170 | Blood | HBsAg - | 17 | 10.0% |
| **Yang et al. 2015** | Taiwan | Others without liver disease/deranged LFTs | "Healthy" controls as in HCC-free, including many HCV +ves in REVEAL study | | 723 | 723 | 723 | Blood | HBsAg - | 53 | 7.3% |
| **Yang et al. 2017** | China | Blood Donors | | Affiliated hospital of Jiangxi University of Traditional Chinese Medicine, Nanchang, China | 1134 | | 255 | Blood | HBsAg -, anti-HBc + | 6 | 2.4% |
| **Yang et al. 2017** | China | Others without liver disease/deranged LFTs | Hyperlipidaemia patients | Affiliated hospital of Jiangxi University of Traditional Chinese Medicine, Nanchang, China | 1036 | | 252 | Blood | HBsAg -, anti-HBc + | 24 | 9.5% |
| **Yazigi et al. 2017** | Syria | Haemodialysis patients | | Al-Assad university hospital, Latakia National Hospital, Latakia Military Hospital , Damascus Kidney Surgical Hospital. | 122 | 122 | 34 | Blood | HBsAg -, anti-HBc + | 4 | 11.8% |
| **Ye et al. 2013** | China | Blood Donors | | Shenzhen blood center, China | 307740 | 307740 | 307740 | Blood | HBsAg - | 80 | 0.0% |
| **Ye et al. 2017** | China | Blood Donors | | Shenzhen Blood Centre | 1033 | 1033 | 490 | Blood | HBsAg -, anti-HBc + | 14 | 2.9% |
| **Ye et al. 2019** | China | Blood Donors | | Shenzhen Blood Center (SZBC) | 259 | 257 | 257 | Blood | HBsAg - | 119 | 46.3% |
| **Ye et al. 2019** | China | Blood Donors | | Shenzhen Blood Center (SZBC) | 123280 | 122552 | 122552 | Blood | HBsAg - | 162 | 0.1% |
| **Yeh et al. 2010** | USA | HCC patients | | University of Washington + Johns Hopkins University | 18 | 18 | 18 | Liver | HBsAg - | 0 | 0.0% |
| **Yilmaz et al. 2013** | Turkey | Others without liver disease/deranged LFTs | Rheumatology patients | Rheumatology Outpatient Clinic | 116 | 116 | 116 | Blood | HBsAg - | 0 | 0.0% |

| | | | | | | | | | | | |
|---|---|---|---|---|---|---|---|---|---|---|---|
| **Yoo et al. 2013** | South Korea | Haemodialysis patients | | HA Bundang Medical Center (Seongnam, Korea) | 98 | 94 | 94 | Blood | HBsAg - | 1 | 1.1% |
| **Yoo et al. 2013** | South Korea | Haemodialysis patients | | HA Bundang Medical Center (Seongnam, Korea) | 98 | 94 | 94 | Blood | HBsAg - | 3 | 3.2% |
| **You et al. 2013** | South Korea | Patients with liver impairment for any other reason | Unclear | Hospital / Health Promotion Centre in Korea (Unspecified) | 14235 | | 571 | Blood | HBsAg -, anti-HBc +ve, anti-HBs - | 27 | 4.7% |
| **Yuan et al. 2010** | China | Blood Donors | | Xiamen Blood Service, Fujian Province, China | 19518 | 19360 | 995 | Blood | HBsAg -, anti-HBc +ve, anti-HBs - | 34 | 3.4% |
| **Yuan et al. 2015** | China | Others without liver disease/deranged LFTs | Patients, not further specified | Chongqing Medical University | 110 | 110 | 110 | Blood | HBsAg - | 26 | 23.6% |
| **YuQiong et al. 2014** | China | Patients with liver impairment for any other reason | Cryptogenic intrahepatic cholangiocarcinoma (ICC) patients from original group randomly selected (above) | Eastern Hepatobiliary Surgery Hospital of the Second Military Medical University (Shanghai, China) | 44 | 44 | 44 | Liver | HBsAg - | 28 | 63.6% |
| **Zhang et al. 2013** | China | General population | | | 121 | 121 | 121 | Blood | HBsAg - | 4 | 3.3% |
| **Zhang et al. 2013** | China | Others without liver disease/deranged LFTs | Healthy controls | | 121 | 121 | 121 | Blood | HBsAg - | 4 | 3.3% |
| **Zhang et al. 2015** | China | Blood Donors | | 14 different hospitals in China, including The First Hospital Affiliated to Anhui Medical University, The Second People's Hospital of Fuyang City, People's Hospital of Luan City, The First People's Hospital of Hefei City, People's Hospital of Taihe County, People's Hospital of Jieshou City, People's Hospital of Chizhou City, People's Hospital of Huaibei City, Mine Workers' General Hospital of Huaibei, People's Hospital of Suixi City, and People's Hospital of Huoqiu County | 579 | 579 | 579 | Blood | HBsAg - | 15 | 2.6% |

| | | | | | | | | | | | |
|---|---|---|---|---|---|---|---|---|---|---|---|
| **Zhang et al. 2017** | China | Blood Donors | | National Center of Clinical Laboratories, Beijing (NCCL) | 2977 | 2795 | 2795 | Blood | HBsAg - | 998 | 35.7% |
| **Zhang et al. 2019** | China | Haematology patients | Haploidentical haematopoietic stem cell transplantation (HSCT) patients | Peking University Institute of Haematology | 227 | 189 | 189 | Blood | HBsAg - | 15 | 7.9% |
| **Zhao et al. 2016** | China | Blood Donors | | | 26374 | 26374 | 26374 | Blood | HBsAg - | 10 | 0.0% |
| **Zhen-Feng et al. 2015** | China | HCC patients | | Fifth Department of Hepatic Surgery, Eastern Hepatobiliary Surgery Hospital, Second Military Medical University (Shanghai, China) | 610 | 106 | 86 | Liver | HBsAg - | 59 | 68.6% |
| **Zheng et al. 2015** | China | Blood Donors | | Shenzhen Blood Center, Shenzhen, China | 2028 | 2028 | 2028 | Blood | HBsAg - | 23 | 1.1% |
| **Zheng et al. 2017** | China | Blood Donors | | Ningxia Hui minority areas | 134495 | 133744 | 133744 | Blood | HBsAg - | 80 | 0.1% |

## References in this document:


1 Polaris Observatory Collaborators. Global prevalence, treatment, and prevention of hepatitis B virus infection in 2016: a modelling study. *Lancet Gastroenterol Hepatol* 2018; **3**: 383–403.

2 Schweitzer A, Horn J, Mikolajczyk RT, Krause G, Ott JJ. Estimations of worldwide prevalence of chronic hepatitis B virus infection: a systematic review of data published between 1965 and 2013. *Lancet* 2015; **386**: 1546–55.

3 World Health Organization. WHO guidelines on hepatitis B and C testing. 2017 http://www.who.int/hepatitis/publications/HEP17001_WEB11.pdf?ua=1 (accessed Dec 5, 2021).

4 Raimondo G, Locarnini S, Pollicino T, *et al.* Update of the statements on biology and clinical impact of occult hepatitis B virus infection. *J Hepatol* 2019; **71**: 397–408.

5 Rehermann B, Ferrari C, Pasquinelli C, Chisari FV. The hepatitis B virus persists for decades after patients' recovery from acute viral hepatitis despite active maintenance of a cytotoxic T-lymphocyte response. *Nat Med* 1996; **2**: 1104–8.

6 Cacciola I, Pollicino T, Squadrito G, Cerenzia G, Orlando ME, Raimondo G. Occult hepatitis B virus infection in patients with chronic hepatitis C liver disease. *N Engl J Med* 1999; **341**: 22–6.

7 Dwamena B. MIDAS: Stata module for meta-analytical integration of diagnostic test accuracy studies. *Statistical Software Components* 2009; published online Feb 5. https://ideas.repec.org/c/boc/bocode/s456880.html (accessed Feb 27, 2022).

8 Servant-Delmas A, Mercier-Darty M, Ly TD, *et al.* Variable capacity of 13 hepatitis B virus surface antigen assays for the detection of HBsAg mutants in blood samples. *J Clin Virol* 2012; **53**: 338–45.

9 Konnick EQ, Erali M, Ashwood ER, Hillyard DR. Evaluation of the COBAS amplicor HBV monitor assay and comparison with the ultrasensitive HBV hybrid capture 2 assay for quantification of hepatitis B virus DNA. *J Clin Microbiol* 2005; **43**: 596–603.

10 Koppelman MHGM, Sjerps MC, Reesink HW, Cuypers HTM. Evaluation of COBAS AmpliPrep nucleic acid extraction in conjunction with COBAS AmpliScreen HBV DNA, HCV RNA and HIV-1 RNA amplification and detection. *Vox Sang* 2005; **89**: 193–200.
